# Assessing anaerobic speed reserve: A systematic review on the validity and reliability of methods to determine maximal aerobic speed and maximal sprinting speed in running-based sports

**DOI:** 10.1371/journal.pone.0296866

**Published:** 2024-01-22

**Authors:** Maximiliane Thron, Peter Düking, Ludwig Ruf, Sascha Härtel, Alexander Woll, Stefan Altmann

**Affiliations:** 1 Institute of Sports and Sports Science, Karlsruhe Institute of Technology, Karlsruhe, Baden-Wuerttemberg, Germany; 2 Department of Sports Science and Movement Pedagogy, Technical University of Braunschweig, Braunschweig, Lower Saxony, Germany; 3 TSG 1899 Hoffenheim, Zuzenhausen, Baden-Wuerttemberg, Germany; 4 TSG ResearchLab gGmbH, Zuzenhausen, Baden-Württemberg, Germany; Università degli Studi di Milano: Universita degli Studi di Milano, ITALY

## Abstract

**Purpose:**

Locomotor profiling using anaerobic speed reserve (ASR) enables insights into athletes’ physiological and neuromuscular contributing factors and prescription of high-intensity training beyond maximal aerobic speed (MAS). This systematic review aimed to determine the validity and reliability of different methods to assess the characteristics of ASR, i.e., MAS and maximal sprinting speed (MSS).

**Methods:**

A comprehensive search of the PubMed and Web of Science databases was conducted according to the PRISMA guidelines. Studies were included if they reported data on validity and/or reliability for methods to assess MAS or MSS.

**Results:**

58 studies were included with 28 studies referring to MAS and 30 studies to MSS. Regarding MAS, different methods for cardiopulmonary exercise testing yielded different values (four out of seven studies) of MAS (Cohen’s d (ES) = 0.83–2.8; Pearson’s r/intraclass correlation coefficient (r/ICC) = 0.46–0.85). Criterion validity of different field tests showed heterogeneous results (ES = 0–3.57; r/ICC = 0.40–0.96). Intraday and interday reliability was mostly acceptable for the investigated methods (ICC/r>0.76; CV<16.9%). Regarding MSS, radar and laser measurements (one out of one studies), timing gates (two out of two studies), and video analysis showed mostly good criterion validity (two out of two studies) (ES = 0.02–0.53; r/ICC = 0.93–0.98) and reliability (r/ICC>0.83; CV<2.43%). Criterion validity (ES = 0.02–7.11) and reliability (r/ICC = 0.14–0.97; CV = 0.7–9.77%) for global or local positioning systems (seven out of nine studies) and treadmill sprinting (one out of one studies) was not acceptable in most studies.

**Conclusion:**

The criterion validity of incremental field tests or shuttle runs to examine MAS cannot be confirmed. Results on time trials indicate that distances adapted to the participants’ sporting background, fitness, or sex might be suitable to estimate MAS. Regarding MSS, only sprints with radar or laser measures, timing gates, or video analysis provide valid and reliable results for linear sprints of 20 to 70 m.

## 1 Introduction

Assessing athletic performance is often performed in sports science and sports practice, e.g. to individualize training procedures. In a variety of team and individual sports and at different performance levels, endurance testing is a required and inevitably part of the training routine, more specifically to set training intensities [1].

Most currently used markers for endurance testing (e.g., lactate thresholds, maximal oxygen uptake (VO_2_max), critical speed) are limited to aerobic performance measures and therefore not applicable for the prescription of training in more intense exercise domains, e.g. intensities above VO_2_max [2]. For this purpose, the anaerobic speed reserve (ASR) as the difference between maximal sprinting speed (MSS) and maximal aerobic speed (MAS) can be used to profile athletes in running type sports on a physiological (referred to MAS) and neuromuscular (referred to MSS) basis and to prescribe training intensities [1, 3]. By using proportions of ASR, exercise intensity beyond MAS can be set by normalizing absolute values of MAS and MSS that allows coaches or researchers to consider the individual tolerance to high-intensity exercise of an athlete [4]. Furthermore, ASR can be used to identify athletes with similar or different characteristics. For example, most team sports require more focus on MSS and thus a higher ASR compared to longer distance events in endurance sports that mostly require a higher MAS and thus lower ASR [1]. However within the same discipline, e.g. in track and field, ASR can differentiate into elite and sub-elite which was indicated by a strong relationship of ASR with 800-m running performance in world class middle-distance runners [3].

To assess ASR, i.e., MAS and MSS, different methods are used that can lead to different results and thus, e.g., different training prescriptions and subsequently adaptations to training [5]. Cardiopulmonary exercise testing (CPET) including breathing gas analysis on a treadmill while employing an incremental protocol is considered as gold standard methodology to assess MAS [6, 7]. MAS can be defined as the first speed associated with VO_2_max [8, 9], yet there is current debate about the exact procedure on how to define this speed. Di Prampero et al. [8] first defined the MAS as a calculated speed based on the ratio of the maximal fraction of VO_2_max and the energy cost of running, intended to describe the speed that a runner can sustain under aerobic conditions. Following, further definitions of MAS emerged as for example the speed at the onset of the VO_2_-plateau and therefore the maximal speed where mainly aerobic resources are used [10, 11], the first speed associated with the 30-s interval of VO_2_max [12, 13], or as the maximal speed reached during the incremental treadmill test [14]. Although even for CPET as the gold standard method different definitions of MAS are used, several field tests are currently implemented to estimate MAS. These range from incremental continuous field tests like the Université the Montréal Track Test (UMTT) or incremental shuttle runs to (set distance) time trials. Consequently, for MAS it is currently unclear which testing profile and which definition of the first speed when reaching VO_2_max is the most valid and reliable.

Regarding MSS, linear sprint tests of 20–50 m with radar or laser measurement are mostly used as gold standard methods [15–18]. However, timing gates with 5- or 10-m split times or Global and Local Positioning Systems (GPS, LPS) are also very common methods. Moreover, GPS and LPS are implemented during matches or training (e.g. small-sided games) mainly in soccer to assess MSS [19].

Since the prescription of high-intensity training and the profiling of athletes based on ASR relies on valid and reliable testing of MAS and MSS to guarantee that any changes are not the result of measurement error (i.e., systematic and random error stemming from technological and biological sources) or intraindividual variances, the selection of measures needs to be carefully considered [20]. While previous reviews focused for example on several influencing factors of sprint performance testing (such as temperature, wind, running surface or shoes) [21] or aerobic fitness tests to assess VO_2_max and often focused on only one type of sports [22], there exists no overview on the validity and reliability of testing methods for MAS and MSS in running-based sports.

Therefore, this review aims to systematically review the available literature on the validity and reliability of different methods to assess the two sub-components of ASR, i.e., MAS and MSS, in running-based sports, e.g., team sports, track and field, and runners at recreational and higher level. The results of this review can be of value for practitioners and scientists to choose the testing methodology that best meets their requirements.

## 2 Methods

This systematic review was written according to the PRISMA (Preferred Reporting Items for Systematic Reviews and Meta-Analyses; see PRISMA 2020 checklist in S1 Table) guidelines [23] and no protocol was registered previously.

### 2.1 Eligibility criteria for included studies

**Fig 1:**
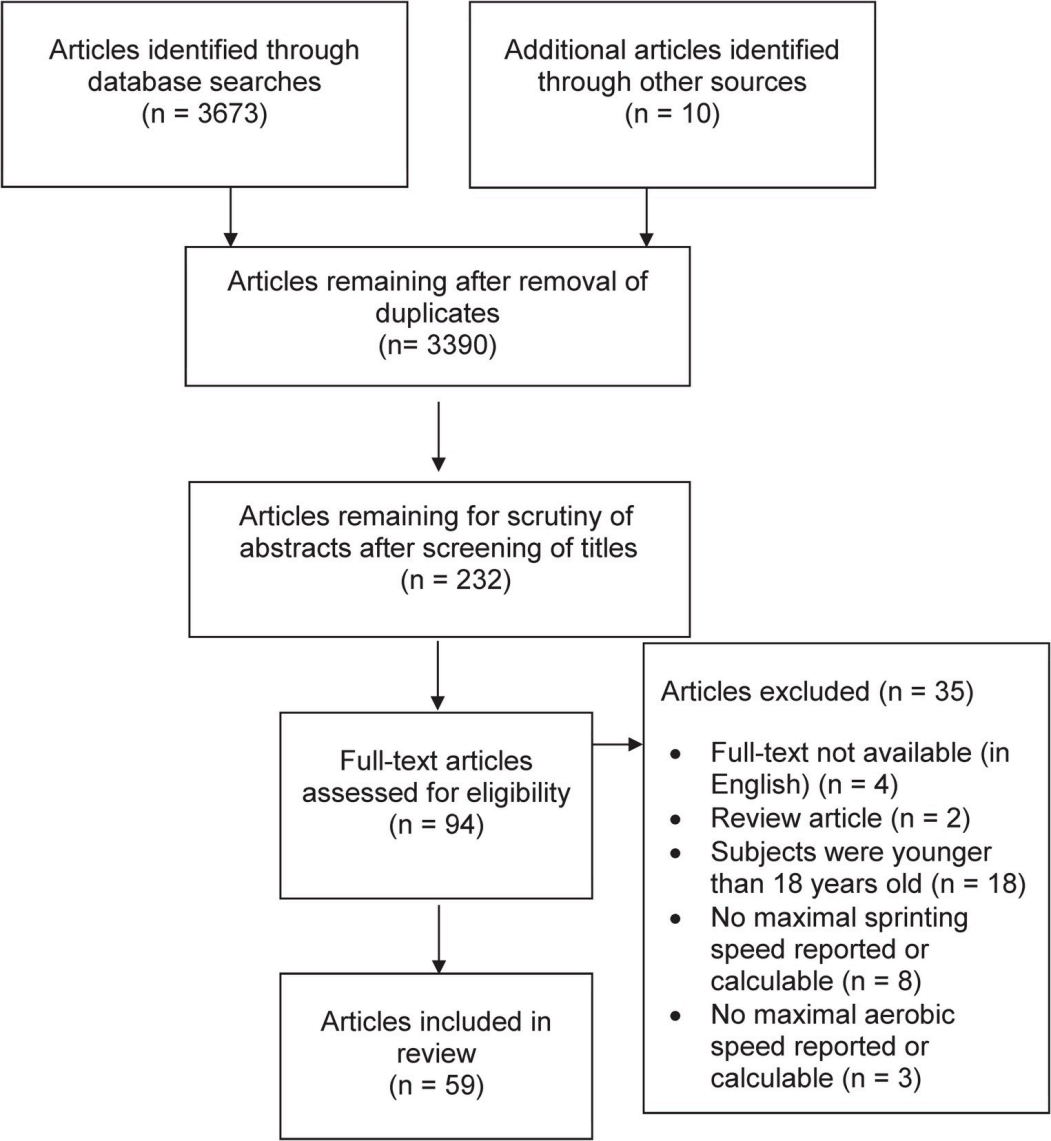
Selection of the articles to be analyzed, from initial identification to inclusion [23].

To be included in the systematic review, the scientifically peer-reviewed publications had to meet the Patient, Intervention, Comparator, Outcome, and Study (PICOS) criteria (comparator criteria not applicable). Our search was limited to original articles published in peer-reviewed journals and written in English. References cited by the articles retrieved were also examined for potential relevance. Conference abstracts, dissertations, theses, and other non-peer-reviewed articles were excluded. Fig 1 illustrates the screening and selection process employed.

#### 2.1.1 Patients

All articles reporting on healthy active adults related to running with no restrictions on sex were included.

#### 2.1.2 Intervention

Studies were included if they specifically evaluated methods to assess MAS and/or MSS. Eligibility criteria for study inclusion consisted of one of the following: (i) tests performed two or more times during one occasion (intraday reliability) or on two or more separate occasions (interday reliability); (ii) compared against other methods (criterion and convergent validity).

#### 2.1.3 Outcome

All outcomes reporting validity and/or reliability of methods to assess MAS and/or MSS were included. If only split times during sprint tests were specified, the speed was calculated based on the time and distance by the authors of the present systematic review.

#### 2.1.4 Study design

Any original comparative studies were included.

### 2.2 Search strategy

A comprehensive search strategy was designed by the authors of this article. The electronic databases searched in August 2022 included PubMed and Web of Science (with no restriction concerning publication date) with an updated search being conducted in December 2022. The search strategy is illustrated using the search terms entered the PubMed database as example (S1 File) and was modified according to the indexing systems of Web of Science.

The following keywords were used to capture validity: validity, logical, criterion, convergent, discrimination, gold standard, level, standard. The following keywords were used to capture reliability: reliability, repeatability, reproducibility, measurement error, consistency, smallest worthwhile change, minimal detectable change, typical error, usefulness, sensitivity, relationship, relation, association, correlation. The following keywords were used to capture MAS: maximal aerobic speed, maximal aerobic velocity, MAS, velocity at VO2max, velocity associated with VO2max, vVO2max. The following keywords were used to capture MSS: maximal sprinting speed, maximal speed, MSS, maximal velocity, peak speed. The following keywords were used to capture ASR: anaerobic speed reserve, anaerobic velocity reserve, ASR.

### 2.3 Selection of studies

The identified articles were incorporated into the systematic review online tool Rayyan (rayyan.ai) where duplicates were eliminated. As previously performed and suggested [23, 24], one of the authors (MT) examined the titles and abstracts of all possibly pertinent papers for eligibility, with independent verification by a second author (SA). The full texts of articles that met the criteria for inclusion were then retrieved and screened. When disagreements between reviewers arose, consensus was achieved through discussion or input from a third author (PD).

### 2.4 Data extraction and analysis

From the selected articles, one author (MT) extracted data which was independently confirmed by another (SA) and difficulties were resolved through discussion with the other authors. Extracted information concerned: details of publication, number of participants, demographic information (including sex, age, type of sports), testing methods with a short test description, reliability and validity type, outcome measures as well as results for validity or reliability, and the information required to assess the methodological quality of each study.

If possible, the mean difference, percentage difference and the respective effect sizes (ES as Cohen’s d) between values of MAS or MSS from different methods or for different sampling time points where retrieved from the studies or calculated manually. ES (Cohen’s d) was rated according to Cohen [25]: less than 0.2 was considered a trivial effect, 0.2≤ES<0.5 small effect, 0.5≤ES<0.8 moderate effect, and ES≥0.8 large effect. Values for the intraclass correlation coefficient (ICC), Pearson’s r and the coefficient of variation (CV) were taken for reliability (both intraday and interday) and validity. CV is a measurement of absolute reliability and validity, whereas ICC and Pearson’s r are indicators of relative reliability and validity. According to Hopkins [26] the magnitude of the correlation was considered to be small (0.1≤r/ICC<0.3), medium (0.3≤r/ICC<0.5), large (0.5≤r/ICC<0.7), very large (0.7≤r/ICC<0.9), and almost perfect (r/ICC≥0.9) classifications. The CV was interpreted in relation to each other [27]. The analysis of the results was done descriptively.

### 2.5 Assessment of methodological quality

Based on the recommendation of Ma et al. [28], the methodological quality of the studies included in this review was assessed through the COnsensus-based Standards for the selection of health Measurement INstruments (COSMIN) checklist by using the boxes for reliability, measurement error, criterion validity, and convergent validity [29, 30]. The risk of bias was assessed independently by two of the authors (PD & MT), with any disagreements again being resolved by consensus or through discussion with a third author (LR) [31].

The score for each item was determined as follows: 3 = *very good*; 2 = *adequate*; 1 = *doubtful*; 0 = *inadequate*, and NA = *not applicable*. The quality of each study was rated with a worst-score-count method to determine the risk of bias [29]. Further evaluation of the methods’ validity (i.e., criterion validity) and recommendations for practical application were based on the studies using an accepted gold-standard method (i.e., CPET for MAS; radar or laser for MSS) and achieving a methodological quality score of at least 2 (*adequate)*.

## 3 Results

### 3.1 Study results and overview of study characteristics

Of the 94 articles initially identified, 59 fulfilled all the criteria for inclusion in the analysis. Of the 29 articles reporting MAS, 23 referred to validity only [6, 7, 10, 12, 32–50], five articles to both validity and reliability [13, 14, 51–53], and one to reliability only [54]. Regarding MSS, 30 studies were included from which 12 studies reported only validity [15, 19, 55–64], two only reliability [17, 65] and 16 both reliability and validity [18, 66–80].

An overview of the study characteristics, including populations’ characteristics, a short description of testing methods, reliability/validity type, and main results is given in Table 1 for MAS and in Table 2 for MSS. Additionally, Table 3 presents a descriptive overview of the participants’ age, the contribution of sexes, and the sporting background.

**Table 1:** Overview of study characteristics and results on the assessment of maximal aerobic speed.

Study	Population				Short description	Type	Results	MQ
	N	Sex	Age [years]	Description				
Barbero-Alvarez et al. [32]	14	Female	21.1 ± 4.0	National team futsal players	CPET on treadmill (protocol: 6-2-3; MAS_calc_: linear function of VO_2_max); Futsal-specific shuttle running test (3 x 15 m with increasing velocities and 10 or 30 s rest; V_ShuttleTest_: peak velocity)	Criterion validity	*Validity*: MAS_calc_ and V_ShuttleTest_: MD = 2.7 km/h (+21.6%); ES = 2.04; r = 0.85	1
Bellenger et al. [33]	28	Male	19.8 ± 2.6	Australian rules football players	UMTT (protocol: 10-1-2; V_UMTT_: maximal velocity); 1.2-km TT (V_1.2km_: average velocity); 1.4-km TT (V_1.4km_: average velocity); 1.6-km TT (V_1.6km_: average velocity); 1.8-km TT (V_1.8km_: average velocity); 2.0-km TT (V_2.0km_: average velocity); 2.2-km TT (V_2.2km_: average velocity)	Convergent validity	*Validity*: V_UMTT_ and V_1200-m_: MD = 1.55 km/h (+9.4%); ES = 2.08; r = 0.70 V_UMTT_ and V_1400-m_: MD = 1.32 km/h (+7.4%); ES = 1.43; r = 0.85 V_UMTT_ and V_1600-m_: MD = 0.45 km/h (+3.5%); ES = 0.82; r = 0.69 V_UMTT_ and V_1800-m_: MD = 0.33 km/h (+1.5%); ES = 0.29; r = 0.81 V_UMTT_ and V_2000-m_: MD = 0.01 km/h (+0.8%); ES = 0.17; r = 0.79 V_UMTT_ and V_2200-m_: MD = -0.29 km/h (-1.8%); ES = 0.33; r = 0.82	3
Benhammou et al. [51]	43	Male	18.2 ± 2.2	Healthy adults of different fitness levels	VAM-EVAL (protocol: 8.5–0.5–1; V_VAM_: peak velocity); Test_3L_ (3/5/7 x running greatest possible distance for 1 minute; depends on fitness level; 30 s active rest; V_3L_: total distance/58 (or 91 or 124; depends on level)	Convergent validity; Interday reliability	*Validity*: Beginners: V_VAM_ and V_3L_: MD = -0.2 km/h (-1.4%); ES = 0.42; r = 0.27 Trained: V_VAM_ and V_3L_: MD = -0.4 km/h (-2.4%); ES = 0.60; r = -0.68 Elite: V_VAM_ and V_3L_: MD = 0.2 km/h (+1.0%); ES = 0.30; r = 0.28 All: V_VAM_ and V_3L_: MD = -0.3 km/h (-1.9%); ES = 0.12; r = 0.93 *Reliability*: Beginners: V_3L_: MD = 0.0 km/h (0%); ES = 0.00; ICC = 0.96; r = 0.97; CV = 5.0% Trained: V_3L_: MD = 0.0 km/h (0%); ES = 0.00; ICC = 0.85; r = 0.87; CV = 2.6% Elite: V_3L_: MD = -0.1 km/h (-0.5%); ES = 0.13; ICC = 0.98; r = 0.99; CV = 3.7% All: V_3L_: MD = 0.1 km/h (0.6%); ES = 0.04; ICC = 0.99; r = 0.99; CV = 16.9%	3 3
Bernard et al. [34]	13	Male	26.0 ± 6.0	Healthy adults	CPET on treadmill: three square-wave tests at speeds differing by 0.5 km/h (highest velocity was based on MAS_30s_ measured with an incremental treadmill test; lowest velocity was MAS_30s_ minus 4 km/h; MAS_4_: extrapolated of VO_2_ at the end of 4^th^ minute; MAS_6_: extrapolated of VO_2_ at the end of 6^th^ minute; MAS_SS_: extrapolated from steady-state VO_2_)	Criterion validity	*Validity*: MAS_SS_ and MAS_6_: MD = 0.24 km/h (+1.3%); ES = 0.30; r = 0.98 MAS_SS_ and MAS_4_: MD = 0.84 km/h (+4.6%); ES = 1.11; r = 0.85 MAS_6_ and MAS_4_: MD = 0.60 km/h (+3.3%); ES = 0.83; r = 0.87	1
Berthoin et al. [35]	11	Male	22.2 ± 3.0	Physical education students	CPET on treadmill (4% incline; protocol: 10-2-4; MAS_max_: velocity of last stage; MAS_calc_: (VO_2_max-0.083)/energy cost (44); MAS_ex_: extrapolated from VO_2_ at each velocity (93); UMTT (protocol: 10-1-2; V_UMTT_: velocity of last stage)	Criterion validity	*Validity*: MAS_max_ and MAS_calc_: MD = -0.15 km/h (-0.8%); ES = 0.06, r = 0.99 MAS_max_ and MAS_ex_: MD = -0.47 km/h (-2.8%); ES = 0.21; r = 0.93 MAS_max_ and V_UMTT_: MD = -0.40 km/h (-2.3%); ES = 0.19; r = 0.96 MAS_calc_ and MAS_ex_: MD = -0.32 km/h (-1.9%); ES = 0.20; r = 0.92 MAS_calc_ and V_UMTT_: MD = -0.25 km/h (-1.5%); ES = 0.17; r = 0.96 MAS_ex_ and V_UMTT_: MD = 0.10 km/h (+0.4%); ES = 0.05; r = 0.85	1
Berthon et al. [36]	48	Male	27.9 ± 6.9	Healthy adults of different fitness levels	CPET on treadmill (1% incline; protocol: 70% of theoretical HR_max_-1.5–3; MAS_max_: maximal velocity); UMTT (protocol: 6.4–0.3–0.5; V_UMTT_: maximal velocity); 5-min TT (V_5min_: average velocity)	Criterion validity	*Validity*: MAS_max_ and V_UMTT_: MD = 1.4 km/h (+8.3%); ES = 0.54; r = 0.95 MAS_max_ and V_5min_: MD = 0.3 km/h (+1.8%); ES = 0.08; r = 0.94	3
Berthon et al. [37]	18 23	Male	Sub-elite runners: 28.7 ± 6.9; Non-running sportsmen: 23.9 ± 5.0	Sub-elite runners; non-running sportsmen	CPET on treadmill (1% incline; protocol: 70% of theoretical HR_max_-1.5–3; MAS_max_: maximal velocity); 5-min TT (V_5min_: average velocity)	Criterion validity	*Validity*: Sub-elite runners: MAS_max_ and V_5min_: MD = 0.1 km/h (+0.5%); ES = 0.11; r = 0.86 Non-running sportsmen: MAS_max_ and V_5min_: MD = 0.2 km/h (+1.3%); ES = 0.17; r = 0.84	3
Billat et al. [6]	15	Male	24.0 ± 2.0	Endurance trained athletes	CPET on treadmill (protocol 1: 15-1-2; protocol 2: 15–0.5–1; MAS_1_/MAS_2_: velocity when VO_2_max occurred as long as velocity was sustained for at least 1 min; if not, then velocity of previous stage was taken); UMTT (protocol: 10-1-2; V_UMTT_: velocity of the last stage)	Criterion validity	*Validity*: MAS_1_ and MAS_2_: MD = 0.1 km/h (+0.5%); ES = 0.11; r = 0.8 MAS_1_ and V_UMTT_: MD = -1.0 km/h (-4.7%); ES = 1.43; r = 0.6	3
Cappa et al. [52]	14	Male	25.5 ± 3.2	Physical education students	CPET on treadmill (protocol: 8 km/h for 3 min, then 10 km/h for 2 min, then 11-1-1; MAS_VO2max_: lowest speed at which VO_2_max was reached); UNCa Test (hexagon of 20-meter-long sides; protocol: 8 km/h for 3 min, then 10 km/h for 2 min, then 11-1-1; V_UNCa_: speed of the last stage)	Criterion validity; Interday reliability	*Validity*: MAS_VO2max_ and V_UNCa_: MD = -2.0 km/h (12.8%); ES = 1.9; r = 0.83 *Reliability*: MAS_VO2max_: MD = -0.1 km/h (-0.7%); ES = 0.11; r = 0.87	3 1
Carminatti et al. [38]	18	Male	21.9 ± 2.0	Physical education students	VAM-EVAL (protocol: 8.5–0.5–1; V_VAM_: peak velocity); CAR test (protocol: 9–0.6–1.5; 6 s active break; V_CAR_: speed of the last stage)	Convergent validity	*Validity*: V_VAM_ and V_CAR_: MD = 0.1 km/h (+0.6%); ES = 0.08; r = 0.98	3
Čović et al. [14]	17	Female	22.8 ± 4.3	Elite soccer players	CPET on treadmill (1.8° incline; protocol: 3 km/h for 3 min, then 7-1-1; MAS_max_: velocity of last stage); 30–15 IFT (protocol: 8–0.5–0.5; 15 s passive break; V_IFT_: velocity of last completed stage)	Criterion validity; Interday reliability	*Validity*: MAS_max_ and V_IFT_: MD = 4.0 km/h (+29.5%); ES = 0.98; r = 0.57 *Reliability*: V_IFT_: MD = 0.3 km/h (+1.6%); ES = 0.29; ICC = 0.91; CV = 1.8%	3 3
Da Silva and Machado [39]	21	Male	41.2 ± 6.9	Amateur runners	CPET on treadmill (protocol: 7 km/h for 3 min; then 9-1-3; MAS_VO2max_: speed in which VO_2_max was observed; MAS_ex_: extrapolated from VO_2_ at each velocity (94); MAS_calc1_: VO_2_max/energy cost of running (8); MAS_calc2_: (VO_2_max-VO_2_rest)/energy cost of running (44); MAS_max_: velocity of last stage)	Criterion validity	*Validity*: MAS_max_ and MAS_VO2max_: MD = -0.1 km/h (-0.7%); ES = 0.07; r = 0.94 MAS_max_ and MAS_calc1_: MD = -0.1 km/h (-0.7%); ES = 0.06; r = 0.91 MAS_max_ and MAS_ex_: MD = -0.4 km/h (-2.6%); ES = 0.27; r = 0.93 MAS_max_ and MAS_calc2_: MD = 0.3 km/h (+2.0%); ES = 0.19; r = 0.91 MAS_VO2max_ and MAS_calc1_: MD = 0.0 km/h (0%); ES = 0.00; r = 0.86 MAS_VO2max_ and MAS_ex_: MD = -0.3 km/h (-2.0%); ES = 0.19; r = 0.90 MAS_VO2max_ and MAS_calc2_: MD = 0.4 km/h (+2.6%); ES = 0.25; r = 0.85 MAS_calc1_ and MAS_ex_: MD = -0.3 km/h (-2.0%); ES = 0.18; r = 0.92 MAS_calc1_ and MAS_calc2_: MD = 0.4 km/h (+2.6%); ES = 0.24; r = 1.00 MAS_ex_ and MAS_calc2_: MD = 0.7 km/h (+4.7%); ES = 0.42; r = 0.92	3
Darendeli et al. [40]	18	Male	18.3 ± 0.5	Amateur soccer players	UMTT (protocol; 10-1-2; V_UMTT_: speed of the last stage); YoYo-IRT 1 (protocol: starting at 10 km/h 40-m shuttles with 10-s active break; V_YoYo-IRT1_: final speed; V_YoYo-IRT1CALC_: 0.701*distance covered+0.03*(height in cm-2.201 (95)); 20-m ST (20-m shuttles; protocol: 8.5–0.5–1; V_20mST:_: final speed; V_20mSTCALC_: V_20mST_ *1.81–7.86) (96)); 5-min TT (V_5min_: average velocity)	Convergent validity	*Validity*: V_UMTT_ and V_YoYo-IRT1_: MD = 1.6 km/h (+10.5%); ES = 1.60; r = 0.69 V_UMTT_ and V_YoYo-IRT1CALC_: MD = 1.0 km/h (+6.5%); ES = 0.72; r = 0.63 V_UMTT_ and V_20mST:_: MD = -2.1 km/h (-13.3%); ES = 2.21; r = 0.70 V_UMTT_ and V_20mSTCALC_: MD = 0.8 km/h (+5.2%); ES = 0.60; r = 0.8 V_UMTT_ and V_5min_: MD = 0.5 km/h (+3.3%); ES = 0.45; r = 0.52	3
Dillon et al. [41]	29	Male	NA	Australian Rules Football	30–15 IFT (protocol: 8–0.5–0.5; 15 s passive break; 80% V_IFT_: 80% of velocity of last completed stage); 3-km TT (V_3km_: average velocity during the 3 km; V_500m_: average velocity during the fastest 500-m split; V_1000m_: average velocity during the fastest 1000-m split)	Convergent validity	*Validity*: 80% V_IFT_ and V_3km_: MD = -0.07 km/h (-0.3%); ES = 0.09; r = 0.72 80% V_IFT_ and V_500m_: MD = 1.37 km/h (+8.1%); ES = 1.65; r = 0.74 80% V_IFT_ and V_1000m_: MD = 1.12 km/h (6.6%); ES = 1.38; r = 0.75	3
Dittrich et al. [42]	30	Male	23.3 ± 4.1	Professional soccer and futsal players	CPET on treadmill (1% incline; protocol: 9–1.2–3 with 0.5 min breaks; MAS_1min_: first speed where VO_2_max occurred and maintained for at least 1 min); CAR test (protocol: 9–0.6–1.5; 6 s active break; V_CAR_: speed of the last stage)	Criterion validity	*Validity*: MAS_1min_ and V_CAR_: MD = -0.7 km/h (-4.1%); ES = 0.32; r = 0.55	3
Foster et al. [43]	22	Male	35.2 ± 8.6	Recreational runners	CPET on treadmill (1% incline; protocol: 3 to 4 speeds for 6 min until last submaximal speed, then 1% elevation increase per minute; MAS_ex_: extrapolation of VO_2_max, (VO_2_max + 27.53)/28.07); 1.61-km TT (V_1.61km_: average velocity; 3.22-km TT (V_3.22km_: average velocity)	Criterion validity	*Validity*: MAS_ex_ and V_1.61km_: MD = 0.97 km/h (+5.6%); ES = 0.51; r = 0.84 MAS_ex_ and V_3.22km_: MD = -0.50 km/h (-3.0%); ES = 0.27; r = 0.86	3
Lacour et al. [44]	24 8	Male Female	23.2 ± 4.6	Experienced runners (800 m–Marathon)	CPET on treadmill (protocol: 10.3–1.54–4 with 1 min breaks; MAS_calc_: (VO_2_max-0.083)/energy cost of running); UMTT (protocol: 6–0.25–0.5; V_UMTT_: velocity during last completed stage); 1500-m TT (V_1500m_: average velocity)	Criterion validity	*Validity*: MAS_calc_ and V_UMTT_: MD = 0.25 km/h (+1.2%); ES = 0.17; r = 0.92 MAS_calc_ and V_1500m_: MD = 1.15 km/h (+5.3%); ES = 0.73; r = 0.90 V_UMTT_ and V_1500m_: MD = 0.90 km/h (+4.1%); ES = 0.59; r = 0.91	3
Laursen et al. [54]	8	Male	31.0 ± 6.0	Experienced runners	Two 5-km TT (V_5km_: average velocity; Two 1.5-km TT c average velocity)	Interday reliability	*Reliability*: V_5km_: MD = 0.22 km/h (1.5%); ES = 0.17; ICC = 0.95; CV = 2.0% V_1.5km_: MD = 0.22 km/h (1.3%); ES = 0.14; ICC = 0.88; CV = 3.3%	3
Lopes et al. [45]	18	Male	28 ± 8	Long distance runners of different levels	CPET on treadmill (protocol 8-1-2; MAS_VO2max_: lowest speed at which VO2max was reached); UMTT (protocol: 8-1-2; V_UMTT1_: velocity of the last completed stage; V_UMTT2_: maximal velocity)	Criterion validity	*Validity*: MAS_VO2max_ and V_UMTT1_: MD = -0.7 km/h (-3.9%); ES = 0.34; ICC = 0.84 MAS_VO2max_ and V_UMTT2_: MD = -0.4 km/h (-2.2%); ES = 0.19; ICC = 0.85	3
Lorenzen et al. [46]	23	Male	22.7 ± 3.4	Australian Rules Football players	CPET on treadmill (1% incline; protocol: 4 km/h below V_3200m_-0.5–1; MAS_VO2max_: lowest speed at which VO2max was reached) 1500-m TT (V_1500m_: average velocity); 3200-m TT (V_3200m_: average velocity)	Criterion validity	*Validity*: MAS_VO2max_ and V_1500m_: MD = 1.30 km/h (+8.0%); ES = 1.75; r = -0.79 MAS_VO2max_ and V_3200m_: MD = -0.61 km/h (-3.7%); ES = 0.83; r = -0.79	3
Lundquist et al. [47]	33	Female	21.3 ± 1.6	Elite Australian Rules Football players	UMTT (protocol: 8-1-2; V_UMTT_: maximal velocity); 1.2-km TT (V_1.2km_: average velocity); 1.4-km TT (V_1.4km_: average velocity); 1.6-km TT (V_1.6km_: average velocity); 1.8-km TT (V_1.8km_: average velocity); 2.0-km TT (V_2.0km_: average velocity); 2.2-km TT (V_2.2km_: average velocity)	Convergent validity	*Validity*: V_UMTT_ and V_1.2km_: MD = 0.6 km/h (+4.6%); ES = 1.09; r = -0.66 V_UMTT_ and V_1.4km_: MD = -0.1 km/h (-0.9%); ES = 0.14; r = -0.96 V_UMTT_ and V_1.6km_: MD = -0.9 km/h (-6.6%); ES = 0.65; r = -0.85 V_UMTT_ and V_1.8km_: MD = -0.6 km/h (-4,7%); ES = 0.83; r = -0.91 V_UMTT_ and V_2.0km_: MD = -0.6 km/h (-4.9%); ES = 0.78; r = -0.94 V_UMTT_ and V_2.2km_: MD = -1.0 km/h (-7.6%); ES = 0.78; r = -0.95	3
Pallarés et al. [48]	22	Male	25.7 ± 7.9	Trained runners and triathletes	CPET on treadmill (1% incline; protocol: 13 km/h less than Vmax during another test-1-1; MAS_VO2max_: lowest speed at which VO_2_max was reached); UMTT (8-1-2; V_UMTT_: last completed stage); track-individualized short ramp graded test (protocol: 13 km/h less than V_max_ from CPET-1-1; V_tracktest_: V_max_*0.8348 + 2.308 (97))	Criterion validity	*Validity*: MAS_VO2max_ and V_UMTT_: MD = -0.5 km/h (-2.6%); ES = 0.42; ICC = 0.92 MAS_VO2max_ and V_tracktest_: MD = 0.0 km/h (+0.3%); ES = 0.00; ICC = 0.94	3
Paradisis et al. [49]	25 23	Male Female	21.2 ± 1.9	Physical education students	CPET on treadmill (1%; protocol: 7–12-1-3; MAS_VO2max_: lowest speed at which VO2max was reached); 20-m ST (20-m shuttles; protocol: 8.5–0.5–1; V_20mSTCALC_: 0.0937*number of shuttles + 6.890)	Criterion validity	*Validity*: MAS_VO2max_ and V_20mSTCALC_: MD = -0.01 km/h (-0.1%); ES = 0.00; r = 0.93	3
Riboli et al. [7]	17	Male	22.6 ± 1.8	Active and healthy adults	CPET on treadmill with a square-wave incremental protocol (1% incline; 5 workloads of 4 min each with at least 5 min rest in between; MAS_ex_: extrapolated from VO_2_ at submaximal velocities); incremental ramp test 1 (1% incline; 10-1-1; MAS1_30s_: first speed where VO_2_max occurred and maintained for at least 30 s); incremental ramp test 2 (1% incline; 10-1-2; MAS2_30s_: first speed where VO_2_max occurred and maintained for at least 30 s)	Criterion validity	*Validity*: MAS_ex_ and MAS1_30s_: MD = 3.9 km/h (+23.2%); ES = 2.8; r = 0.71 MAS_ex_ and MAS2_30s_: MD = 1.9 km/h (+10.7%); ES = 1.7; r = 0.80 MAS1_30s_ and MAS2_30s_: MD = -2.1 (-10.1%); ES = 2.5; r = NA	3
Riboli et al. [50]	16	Male	22.1 ± 1.8	Semi-professional soccer players	CPET on a treadmill with a continuous incremental protocol 1 (1%; 10-1-1; MAS1_30s_: first speed where VO_2_max occurred and maintained for at least 30 s); continuous incremental protocol 2 (1%; 10-1-2; MAS2_30s_: first speed where VO_2_max occurred and maintained for at least 30 s); discontinuous incremental protocol (1%; 5 workloads of 5 min each with at least 5 min rest in between; MAS_ex_: extrapolated from VO_2_ at submaximal velocities)	Criterion validity	*Validity*: MAS1_30s_ and MAS2_30s_ MD = -2.0 km/h (-10.3%); ES = 1.29; r = 0.75 MAS1_30s_ and MAS_ex_: MD = -3.4 km/h (-17.1%); ES = 2.19; r = 0.46 MAS2_30s_ and MAS_ex_: MD = -1.3 (-7.5%); ES = 1.08; r = 0.66	3
Sandford et al. [12]	12 (444)	Male (National junior National senior International junior)	18.5 ± 0.6 22.0 ± 1.8 17.3 ± 0.5	Middle-distance runners	CPET on treadmill (1% incline; 14–18-1-1; MAS_30s_: first speed where VO_2_max occurred and maintained for at least 30 s); 1500-m-TT (V_1500m_: average velocity)	Criterion validity	*Validity*: MAS_30s_: and V_1500m_: MD = 2.06 km/h (9.7%); ES = 1.42; r = 0.90	1
Sangan et al. [13]	26 14 (11 for reliability part)	Male Female	38 ± 7 35 ± 3	Amateur runners	CPET on treadmill (protocol: 7–9–0.5–1; MAS_30s_: first speed where VO_2_max occurred and maintained for at least 30 s); one to three SRT_RPE_ on a running track (three 3-min stages with 1-min rest; intensity at RPE 10, 13, and 17; V_RPE10_: average velocity during last min; V_RPE13_: average velocity during last min; V_RPE17_: average velocity during last min)	Criterion validity; Interday reliability	*Validity*: MAS_30s_: and V_RPE10_: MD = -4.43 km/h (-29.3%); ES = 3.57; r = 0.50 MAS_30s_: and V_RPE13_: MD = -2.66 km/h (-16.5%); ES = 2.24; r = 0.57 MAS_30s_: and V_RPE17_: MD = -0.27 km/h (-2.3%); ES = 0.20; r = 0.66 *Reliability*: V_RPE10_: MD = -0.15–0.15 km/h (-1.4 –+1.4%); ES = 0.14; CV = 5.5%; ICC = 0.76 V_RPE13_: MD = 0.02–0.20 km/h (+0.1–1.5%); ES = 0.19–0.21; CV = 4.5%; ICC = 0.78 V_RPE17_: MD = -0.32–0.04 km/h (-2.1 –+0.3%); ES = 0.03–0.28; CV = 3.9%; ICC = 0.83	3 2
Schnitzler et al. [53]	29 (17 in reliability part)	Male	19 ± 1.3	Physical education students	UMTT (protocol: 8-1-2; V_UMTT_: velocity of last completed stage); two 3´30´´ ECT (protocol: 10 stages of 3 min with 0.5 min rest, first 5 stages at 75% V_UMTT_ and last 5 stages at freely chosen speed to cover maximal distance; V_ECT_: average velocity of last 5 stages)	Convergent validity; Intraday reliability	*Validity*: V_UMTT_ and V_ECT_: MD = -2.6 km/h (-16.5%); ES = 1.27; r = 0.76 *Reliability*: V_ECT_: MD = -0.2 km/h (-1.6%); ES = 0.11; CV = 2.0%; ICC = 0.98	3 2
Thron et al. [10]	13	Male	23.4 ± 2.8	Trained soccer players	CPET on treadmill (1%; protocol: 6-2-3 with 0.5 min rest; MAS_Plateau_: velocity at the onset of the plateau of VO2 (lower increase in VO_2_ than 150 ml/min in at least the last minute of exercise); MAS_30s_: first speed where VO_2_max occurred and maintained for at least 30 s); UMTT (protocol: 10-1-2; V_UMTT_: maximal velocity); 1500-m-TT (V_1500m_: average velocity; V_calc_—V_1500m_*(0.766 + 0.117*1.5 km) (33))	Criterion validity	*Validity*: MAS_Plateau_ and MAS_30s_: MD = 0.99 km/h (+6.3%); ES = 1.61; r = 0.87; ICC = 0.85 MAS_Plateau_ and V_UMTT_: MD = 1.61 km/h (+19.3%); ES = 2.03; r = 0.79; ICC = 0.69 MAS_Plateau_ and V_1500m_: MD = 1.68 km/h (+10.7%); ES = 1.77; r = 0.65; ICC = 0.51 MAS_Plateau_ and V_calc_: MD = 0.67 km/h (+4.3%); ES = 0.70; r = 0.65; ICC = 0.40 V_UMTT_ and V_1500m_: MD = 0.07 km/h (+0.4%); ES = 0.14; r = 0.74; ICC = 0.72	1

The method claimed as the reference method in validity studies is underlined. Criterion validity refers to the comparison with an accepted gold standard method (i.e., CPET on a treadmill for MAS and radar, laser, or motion capture for MSS); convergent validity refers to the comparison with any other method. MQ–Methodological Quality (3 = very good; 2 = adequate; 1 = doubtful; 0 = inadequate); CPET–Cardiopulmonary Exercise Testing; Protocol: starting velocity (km/h)-increment (km/h)-duration of stages (min); MAS–Maximal Aerobic Speed; VO_2_max–Maximal Oxygen Consumption; MD–Mean Difference; ES–Effect Size/Cohen´s d; ICC–Intraclass Correlation Coefficient; CV–Coefficient of Variation; UMTT–Université de Montréal Track Test; TT–Time Trial; VAM-EVAL–Vitesse Aerobie Maximale Evaluation; Test_3L_ –Three Level Intermittent Field Test; HR_max_−Maximal Heart Rate; UNCa test–National University of Catamarca test; CAR test–Carminatti’s Incremental Intermittent Shuttle-Run test; 30–15 IFT– 30-second-15-second Intermittent Fitness Test; YoYo-IRT 1 –YoYo Intermittent Recovery Test 1; 20-m ST– 20-Meter Shuttle Run Test; SRT_RPE_−Self-paced Submaximal Run Test; 3´30´´ ECT– 3-min-30-second Endurance Capacity Test.

**Table 2:** Overview of study characteristics and results on the assessment of maximal sprinting speed.

Study	Population				Short description	Type	Results	MQ
	N	Sex	Age	Description				
Akyildiz et al. [66]	32	Male	21.18 ± 3.16	Amateur soccer players	Eight times a team sport simulation circuit with a 40-m sprint with radar (Stalker ATS II, 50 Hz); GPS (Polar Team Pro; 10 Hz) at chest and back	Criterion validity; Intraday reliability	*Validity*: Radar and GPS chest: MD = 0.03 km/h (+0.1%); ICC = 0.43 Radar and GPS back: MD = 0.31 km/h (+1.3%); ICC = 0.49 *Reliability*: GPS chest: ICC = 0.43; CV = 9.77% GPS back: ICC = 0.49; CV = 9.10%	3 3
Barbero-Alvarez et al. [55]	21	Male	20.2 ± 2.3	Physical education students	Seven 30-m sprints: custom-made infrared timing gates with a 15-m split time (Omron E3S-CR11); GPS at back (SPI Elite, GPSports: 1 Hz)	Convergent validity	*Validity*: Light sensor and GPS: r = -0.93	1
Beato et al. [18]	20	Male	21.0 ± 2.0	Students	Two 20-m-sprints with Radar (Stalker ATS II; 34.7 Hz); GPS at back (Viper; STATSports; 10 Hz)	Criterion validity; Interday reliability	*Validity*: Radar and GPS: MD = 0.27 km/h (+0.7%); ES = 0.07; r = 0.98 *Reliability*: GPS: MD = 0.24 km/h; ES = 0.09; CV = 0.7%	3 1
Beato et al. [67]	10	Male	22.0 ± 1.0	Team sports players	30-m sprint with 5-m split times and GPS: GPS at back (Apex; STATSports; 10 Hz); GPS at back (Viper; STATSports; 10 Hz)	Convergent validity; Intraday reliability	*Validity*: Apex and Viper: MD = 0 km/h (0%); ES = 0.00; ICC = 0.96 *Reliability*: Apex: MD = -0.07 km/h (-0.3%); ES = 0.03; ICC = 0.97; CV = 2.64% Viper: MD = 0.04 km/h (+0.1%); ES = 0.02; ICC = 0.91; CV = 2.62% *On average*, *MSS was reached at the 20–30-m interval*	3 1
Chahal et al. [68]	4 9	Male Female	21.6 ± 1.6	Healthy active adults	One 45.72-m sprint with two smartphone video cameras (Kinovea; 60 Hz); 8 GPS units at back (Catapult S5 OptimEye; 10 Hz)	Convergent validity; Intraday reliability	*Validity*: Video and GPS: MD = -3.31–2.23 km/h (-11.8–8.0%); ES = 0.03–0.78 *Reliability*: GPS: MD = 5.54 km/h (+22.4%); ES = 0.76; ICC = 0.32; CV = 7.01%	0 3
Clark et al. [56]	260	Male	NA	Professional football players	36.6-m sprints with split times at 9.1 and 18.3 m collected via online sources (MSS modelled via a mono-exponential equation)	Convergent validity	*Validity*: 0–9.1 m and modelled MSS: MD = 13.07 km/h (+40.0%); r = 0.85 9.1–18.3 m and modelled MSS: MD = 3.70 km/h (+11.3%); r = 0.69 18.3–36.6 m and modelled MSS: MD = -0.09 km/h (-0.3%); r = 0.97	1
Coutts and Duffield [69]	2	Male	32 ± 2	Moderately trained adults	Eight times a 128.5-m running circuit with a 20-m sprint and GPS: 6 GPS (2 SPI-10; 2 SPI Elite; 2 WiSPI; GPSports; 1 Hz)	Convergent validity; Intraday reliability	*Validity*: SPI-10 and SPI Elite: MD = 0.60 km/h (+2.8%), ES = 0.32; r = 0.40–0.53 SPI-10 and WiSPI: MD = 0.40 km/h (+1.9%); ES = 0.21; r = 0.40–0.53 WiSPI and SPI Elite: MD = 0.20 km/h (+0.9%); ES = 0.11; r = 0.40–0.53 *Reliability*: SPI-10: CV = 5.8% SPI Elite: CV = 2.3% WiSPI: CV = 4.9%	0 0
Djaoui et al. [19]	48	Male	22.6 ± 2.75	Professional and higher amateur soccer players	Three 40-m sprints with GPS (GPSports SPI Elite; 15 Hz); 6 official matches (GPSports SPI Elite; 15 Hz); 14 Small-sided games of 90 min (GPSports SPI Elite; 15 Hz)	Convergent validity	*Validity*: 40-m sprint and matches: MD = -2.35 km/h (-7.5%); ES = 1.07; r = 0.52 40-m sprint and small-sided games: MD = -7.64 km/h (-24.4%); ES = 3.17; r = NA Small-sided games and matches: MD = -5.29 km/h (-18.3%); ES = 1.82; r = NA	0
Ferro et al. [57]	42	Male	21.14 ± 1.62	Physical Activity and Sports Sciences students	Three 30-m sprints with a laser sensor and with 10-m splits (LDM301; Jenoptik; 2000 Hz)	Criterion validity	*Validity*: Average velocities: 20–30 and 0–10 m: MD = -10.04 km/h (-33.4%); ES = 9.69 20–30 and 10 – 20m: MD = -1.76 km/h (-5.1%); ES = 1.44 Maximum velocities: 20–30 and 0–10 m: MD = -3.38 km/h (-11.4%); ES = 3.88 20–30 and 10–20 m: MD = -1.66 km/h (-5.2%); ES = 1.30 *Most players reached MSS in 20-30-m split*	0
Fleureau et al. [58]	5	Male	29.2 ± 4.1	Recreational handball players	Two 25-m sprints with a 12-camera Vicon motion capture system with one marker at the upper back (Vicon Nexus Z40; 250 Hz); 14 LPS antennas and one tag at the upper back (Kinexon; 20 Hz); carried out at the center and at the side of a handball field	Criterion validity	*Validity*: Center of court: Motion capture and LPS: MD = 0.54 km/h (+2.9%); ES = 0.50; r = 1.00 Side of court: Motion capture and LPS: MD = 0.61 km/h (+2.8%); ES = 1.00; r = 0.97	0
Fornasier-Santos et al. [70]	13 5	Male Female	27.0 ± 6.7	Healthy, active adults	Three 40-m sprints with a linear motorized encoder (1080 Sprint; 333 Hz); Laser Speed device (MuscleLab; 1000 Hz); Radar (Stalker Pro II Sports Radar Gun; 46.875 Hz); GPS at the upper back (Vector S7; Catapult; 10 Hz); Timing gates at 0, 5, 10, 15, 20, 30, 40 m (Witty Microgate)	Convergent validity; Intraday reliability	*Validity*: Linear encoder and laser: MD = -0.11 km/h (-0.4%); ES = 0.03 Linear encoder and radar: MD = -0.07 km/h (-0.2%); ES = 0.02 Linear encoder and GPS: MD = 0.00 km/h (0%); ES = 0.00 Linear encoder and timing gates: MD = 0.07 km/h (+0.2%); ES = 0.02 Laser and radar: MD = 0.04 km/h (+0.1%); ES = 0.02 Laser and GPS: MD = 0.11 km/h (+0.4%); ES = 0.03 Laser and timing gates: MD = 0.18 km/h (+0.6%); ES = 0.04 Radar and GPS: MD = 0.07 km/h (+0.3%); ES = 0.02 Radar and timing gates: MD = 0.14 km/h (+0.6%); ES = 0.04 GPS and timing gates: MD = 0.07 (+0.2%); ES = 0.02 *Reliability*: Linear encoder: MD = 0.14 km/h (+0.5%); CV = 1.13% Laser: MD = 0.18 km/h (+0.6%); CV = 1.11% Radar: MD = 0.29 km/h (+1.0%); CV = 1.37% GPS: MD = 0.25 km/h (+0.9%); CV = 1.47% Timing gates: MD = 0.14 km/h (+0.5%); CV = 1.31%	0 0
Ghigiarelli et al. [71]	16 6	Female Male	20.1 ± 1.5	Division II university team sports athletes	Three 30-m sprints with a laser speed device (MuscleLab 6000 ML6LDU02; 2.5 Hz); Video analysis (MySprint app; Apple Inc.; 120 fps)	Criterion validity, Interday reliability; Intraday reliability	*Validity*: Laser and video: MD = 1.12 km/h (+4.0%), ES = 0.53; r = 0.98; ICC = 0.92 *Reliability*: Intraday: Laser: MD = 0.04–0.07 km/h (+0.1–0.3%); ES = 0.02–0.08; ICC>0.98; CV<1.2% Intraday: Video: MD = NA; ES = NA; ICC>0.97; CV<1.6% Interday: Laser: MD = -0.18 km/h (-0.6%); ES = 0.09; ICC = 0.98; CV = 1.1%	3 3 3
Helland et al. [65]	17	Female	23 ± 3	Elite handball players	Two 30-m sprints with timing gates (NA) at 5, 10, 15, 20, and 30 m and mono-exponential modelled MSS	Intraday reliability	*Reliability*: MSS: MD = -0.14 km /h; CV = 1.4%; r = 0.99	1
Highton et al. [72]	12	Male	22.3 ± 3.6	Amateur team sport players	Three 30-m sprints with timing gates and 10-m splits (Speedtrap II, Brower Timing Systems); two times three 30-m sprints on a NMT with a strain gauge (Woodway, Force 3.0; 100 Hz) on two different days	Convergent validity; Interday reliability, Intraday reliability	*Validity*: 0–10 m; overground: and NMT: MD = -6.20 km/h (-40.6%); ES = 3.72; r = 0.43 10–20 m; overground and NMT: MD = -7.70 km/h (-38.5%); ES = 5.85; r = 0.50 20–30 m; overground and NMT: MD = -10.33 km /h (-52.5%); ES = 7.11; r = 0.67 *Reliability*: Interday: NMT: MD = -0.07 km/h (-0.4%); ES = 0.07; CV = 1.8% Intraday: NMT: MD = -0.11–0.07 km/h (-0.5–0.4%); ES = 0.08–0.10; CV = 1.2% *Most subjects reached MSS during the 20–30 m split during the overground running*.	3 0 0
Hoppe et al. [73]	6	Male	27 ± 2	Team sport players	Ten times a team sport simulation circuit with a 30-m sprint with timing gates at 5, 10, 20, and 30 m (Werthner Sport Consulting; TDS); GPS at back (Catapult: 10 Hz); GPS at back (Exelio srl; GPEXE PRO; 18 Hz); LPS at back with 12 antennas (Kinexon; 20 Hz); MSS was modelled mono-exponentially	Convergent validity, Intraday reliability	*Validity*: Timing gates and 10 Hz GPS: MD = 0.0 km/h (0%); ES = 0.00 Timing gates and 18 Hz GPS: MD = 0.0 km/h (-0.2%); ES = 0.00 Timing gates and 20 Hz LPS: MD = 0.72 km/h (+2.1%); ES = 2.00 *Reliability*: 10 Hz GPS: MD = -0.36 km/h (-1.2%); ES = 1.0; CV = 3.3% 18 Hz GPS: MD = 0.0 km/h (0%); ES = 0.0; CV = 3.1% 20 Hz LPS: MD = 0.36 km/h (+1.2%); ES = 1.0; CV = 1.6%	0 0
Johnston et al. [75]	4	Male	29.0 ± 5.0	Well-trained adults	Ten 50-m- sprints with radar (Stalker ATS; 31.25 Hz); Two GPS units at the upper back (Catapult MinimaxX; Team Sport 2.5; 5 Hz)	Criterion validity; Intraday reliability	*Validity*: Radar and GPS 1: r = 0.46 Radar and GPS 2: r = 0.36 *Reliability*: GPS: ICC = 0.01	0 3
Johnston et al. [74]	8	Male	26.1 ± 4.1	Trained adults	Eight laps of a team sports simulation circuit with a 30-m sprint with timing gates and 10-m split times (Swift Performance); Two GPS units (Catapult MinimaxX S4; 10 Hz); Two GPS units (SPI-ProX; GPSports; 15 Hz)	Convergent validity; Intraday reliability	*Validity*: Timing gates and MinimaxX: MD = 0.51–0.52 km/h (+2.3%); ES = 0.22; r = 0.89–0.91 Timing gates and GPSports: MD = -0.51–0.04 km/h; ES = 0.02–0.27 (-2.1–0.1%); r = 0.64–0.76 MinimaxX and GPSports: MD = -0.19 km/h (-0.8%); ES = 0.13 *Reliability*: Inter-unit: MinimaxX: MD = 0.01 km/h (0%); ES = 0.0; ICC = 0.97 Inter-unit: GPSport: MD = -0.55 km/h (-2.3%); ES = 0.3; ICC = -0.14	0 3
Lacome et al. [15]	5	Male	25 ± 5	Elite rugby seven players	Five 40-m sprints with radar (Stalker ATS II; 48 Hz); GPS at the upper back (Sensoreverywhere V2; Digital simulation; 16 Hz)	Criterion validity	*Validity*: Radar and GPS: MD = -0.94 km/h (-3.0%); ES = 0.78 *Reliability*: -	0
Massard et al. [59]	23	Male	21.4 ± 3.8	Semi-professional football players	One 40-m sprint with dual-beamed timing gates and 10-m split times (TT Wireless SpeedLight); GPS at upper back (MinimaxX S4, Catapult 10 Hz) during sprint and matches in preseason and competitive season	Convergent validity	*Validity*: Timing gates and GPS during sprint: MD = -0.3 km/h (-1.0%); ES = 0.21; r = 0.84 Timing gates and GPS during matches: MD = 0.8 km/h (+1.9%); ES = 0.39; r = NA GPS during sprint and GPS during matches: MD = 1.1 km/h (+3.0%); ES = 0.66; r = NA	0
Morin and Sève [60]	11	Male	24.4 ± 3.9	Physical education students	One 100-m sprint in the field with radar (Stalker ATS; 35 Hz); One 100-m sprint on a motorized instrumented treadmill with piezoelectric force transducers (ADAL3D-WR; Medical Development; 1000 Hz); MSS was modelled bi-exponentially	Criterion validity	*Validity*: Radar and treadmill: MD = -6.98 km/h (-21.9%); ES = 4.27; r = 0.89	1
Ogris et al. [61]	6	Male	22 ± 4	Moderately trained soccer players	Six 26.5-m sprints with a motion capture system with 8 cameras (Vicon; 50 Hz); LPS with 12 antennas and the transponder worn on the back (lpm04.59; 45.45 Hz)	Criterion validity	*Validity*: Motion capture and LPS: MD = 1.68 km/h (+6.78%)	0
Roe et al. [62]	9	Male	19.0 ± 0.8	Professional rugby union player	Three 40-m sprints with radar (Stalker ATS II; 50 Hz); Timing gates with 10-m split times (Brower Timing Systems); Three GPS units on the back (Optimeye S5; Catapult; 10 Hz)	Criterion validity	*Validity*: Radar and GPS: MD = 0.25–0.50 km/h (-0.77 - -1.35%); ES = 0.13–0.25; r>0.95 Radar and timing gates: MD = -0.76 km/h (-2.39%); ES = 0.37; r = 0.97	0
Romero-Franco et al. [76]	12	Male	21.4 ± 3.9	Trained sprinters	Six 40-m sprints with radar (Stalker ATS II; 46.88 Hz); Video analysis with split times at 5, 10, 15, 20, and 30 m (MySprint App, Apple Inc.)	Criterion validity; Intraday reliability	*Validity*: Radar and video: MD = 0.25 km/h (+0.8%); ES = 0.07; r = 0.99; ICC = 0.99 *Reliability*: Video: CV = 0.14% Radar: CV = 0.11%	1 0
Sagiroglu et al. [77]	16	Male	27.22 ± 4.70	Amateur soccer players	Three 40-m sprints with radar (Stalker ATS II; 50 Hz); Two GPS-units on the chest (Polar Team Pro GPS; 10 Hz)	Criterion validity; Intraday reliability	*Validity*: Radar and GPS: MD = 0.14 km/h (+1.6%); ES = 0.02; r = 0.94 *Reliability*: GPS: CV = 5–6% Radar: CV = 6%	3 0
Simperingham et al. [17]	27 (9 for Interday reliability)	Male	18.6 ± 0.6	Amateur rugby union players	Three 30-m sprints with radar on four different days (Stalker ATS II; 47 Hz)	Intraday reliability; Interday reliability	*Reliability*: Intraday: CV = 1.8%; ICC = 0.95 Interday: CV = 2.1–2.4%; ICC = 0.83–0.93	3 3
Vescovi [63]	140	Female	23.9 ± 2.8	Professional soccer players	Two 35-m sprint with timing gates and split times at 5, 10, and 20 m (Brower Timing Systems)	Convergent validity	*Validity*: 0–5 m and 5–10 m: MD = 2.9 km/h (+19.2%); ES = 2.89 5–10 m and 10–20: MD = 3.2 km/h (+17.8%); ES = 3.56 10–20 m and 20–35 m: MD = 2.2 km/h (+10.4%); ES = 2.44 *Reliability*: - *Most players reached MSS in the 20–35 m interval*.	0
Willmott et al. [78]	14	Male	23 ± 3	Moderately-trained adults	Two 40-m sprints and a team sports simulation circuit with a 20-m sprint and two different GPS devices (FieldWiz; UNA Sports Medicine; 10 Hz; Catapult MinimaxX S4; Catapult Innovations; 10 Hz)	Convergent validity; Intraday reliability	*Validity*: 40-m sprint: FieldWiz and MinimaxX: MD = -0.3–0.1 km/h (-1.2 - +0.4%); ES = 0.05–0.23; ICC>0.8 40-m sprint vs. 20-m sprint during circuit: MinimaxX: MD = -3.0 km/h (-10.8%); ES = 2.04; ICC>0.8 FieldWiz: MD = -2.6 km/h (-9.3%); ES = 1.41; ICC>0.8 *Reliability*: FieldWiZ: MD = 0.1 km/h (+0.4%); ES = 0.05; CV = 0.9–2.3%; ICC>0.9	1 3
Young et al. [64]	35 (A) 30 (B)	Male	NA	Professional Australian Rules Football players	Club A: 40-m sprint with split times at 10 and 20 m and timing gates (Swift Timing); Club B: 30-m sprint with split times at 10 and 20 m and timing gates (KMS; Fitness Technologies)	Convergent validity	*Validity*: Club A: 0–10 m and 10–20 m: MD = 9.95 km/h (+52.4%); r = 0.94 10–20 m and 20–40 m: MD = 2.55 km/h (+8.8%); r = 0.67 Club B: 0–10 m and 10–20 m: MD = 7.85 km/h (+37.0%); r = 0.94 10–20 m and 20–30 m: MD = 2.00 km/h (+6.7%); r = 0.77	3
Zabaloy et al. [79]	19	Male	22.5 ± 5.3	Amateur rugby players	Two 30-m sprints with radar (Stalker ATS II; 47 Hz); timing gates with split times at 5, 10, 20, and 25 m (Witty System; Microgate)	Criterion validity; Intraday reliability	*Validity*: Radar and best 5-m-split: MD = 0.13 km/h (+0.5%); ES = 0.07; r = 0.93; ICC = 0.96 Radar and best 10-m-split: MD = -0.30 km/h (-1.0%); ES = 0.14; r = 0.97; ICC = 0.98 *Reliability*: Radar: MD = -0.11 km/h (-0.4%); CV = 1.14%; ICC = 0.99 Best 10-m-split: MD = -0.04 km/h (-0.1%); CV = 1.47%; ICC = 0.98 Best 5-m-split: MD = -0.11 km/h (-0.4%); CV = 1.70%; ICC = 0.97 *No significant difference in the 20–25 and 25-30-m-splits*, *indicating that subjects reached MSS between 20 and 30 m*.	3 3
Zabaloy et al. [80]	49	Male	24.16 ± 4.08	Amateur rugby players	Two 50-m sprints with timing gates and split times at 10, 20, 30, and 40 m (Witty System, Microgate)	Validity; Intraday reliability:	*Validity*: 10–20 m and 20–30 m: MD = 1.04–2.09 km/h (0 - +35%); ES = 0.62–0.98 20–30 m and 30–40 m: MD = -0.25–0.22 km/h (0 - +31.1%); ES = 0.07–0.08 30–40 m and 40–50 m: MD = -0.24–0.65 km/h (0–32.9%); ES = 0.11–0.26 *Reliability*: 10-m-split: CV = 2.43%; ICC = 0.95 20-m-split: CV = 2.19%; ICC = 0.97 30-m-split: CV = 1.93%; ICC = 0.98 40-m-split: CV = 1.90%; ICC = 0.98 50-m-split: CV = 2.26%; ICC = 0.98 *50–58*.*6% of the players reached their MSS between 20 and 30 m or between 30 and 40 m*.	0 2

The method claimed as the reference method in validity studies is underlined. Criterion validity refers to the comparison with an accepted gold standard method (i.e., CPET on a treadmill for MAS and radar, laser, or motion capture for MSS); convergent validity refers to the comparison with any other method. MQ–Methodological quality (3 = very good; 2 = adequate; 1 = doubtful; 0 = inadequate); MD–Mean difference; ES–Effect size/Cohen´s d; ICC–Intraclass Correlation Coefficient; CV–Coefficient of variation; GPS–Global Positioning System; Hz–Hertz; MSS–Maximal Sprinting Speed; LPS–Local Positioning System; NA–Not Available; NMT–Non-Motorized Treadmill.

**Table 3:** Descriptive overview.

	Participants [number (mean ± SD; range)]	Age [mean ± SD (range)]	Distribution of sexes [number of studies]		Backgrounds [number of studies]	
MAS	693 (20.8±9.1; 8–48)	24.9 ± 5.9 years (18.3–41.2 years)	Men: n = 23 Women: n = 3 Both: n = 3	[6, 7, 10, 12, 33–43, 45, 46, 48, 50–54] [14, 32, 47] [13, 44, 49]	Soccer/football/futsal players: n = 10 Sports students: n = 5 Runners: n = 10 Healthy active people: n = 4	[10, 14, 32, 33, 40–42, 46, 47, 50] [35, 38, 49, 52, 53] [6, 12, 13, 37, 39, 43–45, 48, 54] [7, 34, 36, 51]
MSS	936 (27.5 ± 47.9; 2–260)	23.5 ± 3.3 years (18.6–31.0 years)	Men: n = 25 Women: n = 2 Both: n = 3	[15, 17–19, 55–62, 64, 66, 67, 69, 72–80] [63, 66] [68, 70, 71]	Soccer/football/rugby/handball: n = 19 Healthy active people: n = 6 Sports students: n = 4 Runners: n = 1	[15, 17–19, 56, 58, 59, 61–66, 71–73, 77, 79, 80] [68–70, 74, 75, 78] [55, 57, 60, 67] [76]

MAS–Maximal Aerobic Speed; MSS–Maximal Sprinting Speed; SD–Standard Deviation.

### 3.2 Assessment of methodological quality

The validity of methods to assess MAS was reported in 28 studies using 69 different methods. For 23 studies the methodological quality for validity was rated *very good* [6, 7, 13, 14, 33, 36–53] and for five studies *doubtful* [10, 12, 32, 34, 35]. Intraday (one method) and interday reliability (six methods) of MAS was reported in one and five studies, respectively. The methodological quality of the study reporting intraday reliability was rated *adequate* [53]. For interday reliability, two studies were rated *very good* [14, 51, 54], one was *adequate* [13] and one was *doubtful* [52].

Regarding MSS, validity was reported in 28 studies using 53 different methods. Methodological quality was rated as *very good* (n = 8) [18, 64, 66, 67, 71, 72, 77, 79], *doubtful* (n = 5) [55, 56, 60, 76, 78], and as *inadequate* (n = 15) [15, 19, 57–59, 61–63, 68–70, 73–75, 80]. Intraday and interday reliability was assessed in 17 and four studies (for 32 and four methods, respectively). Studies reporting intraday reliability achieved a rating for methodological quality of *very good* (n = 8) [17, 66, 68, 71, 74, 75, 78, 79], *adequate* (n = 1) [80], *doubtful* (n = 2) [18, 65], and *inadequate* (n = 6) [69, 70, 72, 73, 76, 77]. Regarding interday reliability, studies were rated as *very good* (n = 2) [17, 71], *doubtful* (n = 1) [67], and *inadequate* (n = 1) [72]. More detailed information about the methodological quality of the studies can be found in S2 Table.

### 3.3 Main findings for criterion validity and reliability

#### 3.3.1 Maximal aerobic speed

*Reference methods used for validity testing*. As a reference method for validity testing, MAS was assessed by CPET on a treadmill in 21 studies (criterion validity), by incremental continuous field tests (UMTT or Vitesse Aerobie Maximale Evaluation (VAM-EVAL)) in six studies, and by the 30–15 Intermittent Fitness Test (30–15 IFT) in one study (convergent validity).

*Cardiopulmonary exercise testing*. Different protocols for CPET where compared with each other seven times. In terms of validity, the results differed between 0.1 and 3.9 km/h (ES = 0.11–2.8; r/ICC = 0.46–0.80). Intraday or interday reliability was not reported for different treadmill protocols. Different definitions of MAS where compared 12 times with deviations of 0–0.99 km/h (ES = 0–1.61; r/ICC = 0.85–1.0). Interday reliability of definitions for MAS was reported one time with a mean difference of -0.1 km/h (ES = 0.11; r/ICC = 0.87), while intraday reliability was not reported.

*Incremental field tests*. Validity of incremental continuous field tests (UMTT, VAM-EVAL, track-individualized short ramp graded test, or National University of Catamarca test) to estimate MAS were tested 12 times against CPET on a treadmill as the reference method. The mean difference between the estimated MAS and the MAS retrieved by CPET ranged from -2.0–1.61 km/h (ES = 0–2.03; r/ICC = 0.83–0.96). Intraday or interday reliability was not reported for incremental continuous field tests.

*Time trials*. Validity for time trials (1500 m, 3200 m, or 5 min) to estimate MAS was examined nine times. The mean difference from the gold standard, i.e., CPET, ranged from 0.1–2.06 km/h (ES = 0.08–1.75; r/ICC = 0.51–0.94). The interday reliability of time trials was reported as good in one study with a mean difference of 0.22 km/h (ES = 0.14–0.17; ICC = 0.88–0.95; CV = 2.0–3.3%).

*Shuttle runs*. Results from different shuttle runs (futsal specific shuttle test, 30–15 IFT, Carminatti’s shuttle test, 20-m shuttle test) to estimate MAS were compared to CPET four times for validity testing. The mean differences ranged from -0.7–4.0 km/h (ES = 0–2.04) with a correlation between 0.55 and 0.93. Regarding interday reliability, one study reported results for the 30–15 IFT with a mean difference of 0.3 km/h (ES = 0.29; ICC = 0.91; CV = 1.8%). Intraday reliability from one study for the 3-min 30-second Endurance Capacity Test showed a mean difference of -0.2 km/h (ES = 0.11; ICC = 0.98; CV = 2.0%).

*Other/individualized methods*. In two studies, validity of other fitness tests to estimate MAS were investigated. The comparison of the self-paced submaximal running test in which the velocities of the stages were self-chosen according to ratings of perceived exertion (RPE) 10, 13, and 17 with CPET showed mean differences between -0.27 and -4.43 km/h (ES = 0.20–3.57; r/ICC = 0.50–0.66). Interday reliability was reported for each test (n = 2). Results for the mean differences were -0.32–0.20 km/h (ES = 0.03–0.28; r/ICC = 0.76–0.99; CV = 3.9–16.9%). Intraday reliability was not examined.

#### 3.3.2 Maximal sprinting speed

*Reference methods used for validity testing*. As a gold standard method to assess MSS, 20–100-m linear sprints with radar or laser measurement were used in 11 studies investigating validity. 30–40-m sprints with 5- or 10-m split times and timing gates were used in eight studies, a 40-m sprint with GPS measurement was used in four studies, 25–26.5-m sprints with a motion capture system were used in two studies, a 45.72-m sprint with video analysis via a smartphone app was used in one study, and a 40-m sprint with a linear encoder was also used in one study as a reference method to assess MSS. In one study, data were collected via online-sources and the reference MSS was modelled mono-exponentially based on the data.

*Radar/Laser technology*. In one study validity of radar and laser measurement was tested with a linear encoder as the reference method. The results differed between -0.11 and -0.07 km/h (ES = 0.02–0.03). Intraday reliability was reported four times and ranged from 0.04–0.29 km/h (ES = 0.02–0.08; ICC>0.95; CV<1.8%). Interday reliability was reported two times for radar/laser measurement with a mean difference of -0.18 km/h (ES = 0.09; ICC = 0.83–0.98; CV<2.4%).

*Timing gates*. In two studies validity for the assessment of MSS with timing gates was examined with radar as the reference method. The results differed between -0.13 and -0.76 km/h (ES = 0.07–0.37; r/ICC = 0.93–0.98). In four studies, 5- or 10-m split times during 30–50-m sprints were compared with each other, resulting in differences between -0.06 and 9.95 km/h (ES = 0.07–3.56; r/ICC = 0.67–0.94). Intraday reliability for measurements of MSS with timing gates was reported five times. Results ranged from -0.14–0.14 km/h (r/ICC = 0.95–0.99; CV = 1.14–2.43%). Interday reliability was not reported.

*Global and local positioning systems*. Validity of GPS or LPS to assess MSS was examined eight times with radar measurement. The results differed by values between 0.03 and 0.94 km/h (ES = 0.02–0.78; r/ICC = 0.36–0.98). GPS/LPS was compared four times with video analysis or motion capture systems. The results differed between -3.31 and 2.23 km/h (ES = 0.03–1.00; r/ICC = 0.97–1.00). Intraday reliability of GPS/LPS measurements to assess MSS during linear sprinting was examined 12 times with results differing between -0.36 and 5.54 km/h (ES = 0–1.0; r/ICC = 0.14–0.97; CV = 0.7–9.77%). Interday reliability was examined one time with a mean difference of 0.24 km/h (ES = 0.09; CV = 0.7%).

*Video analysis via smartphone applications*. Validity of video analysis with a smartphone app was examined two times with radar/laser measurement. The results differed between 0.25 and 1.12 km/h (ES = 0.07–0.53; r/ICC = 0.92–0.99). Intraday reliability was determined two times with an ICC>0.97 and CV<1.6%. Interday reliability was not examined.

*Treadmill sprinting*. Validity of a sprint on a motorized treadmill with piezoelectric force transducers with radar as the reference on a running track was determined one time. The results showed a mean difference of -6.98 km/h (ES = 4.27; r = 0.89). Interday and intraday reliability for the non-motorized treadmill was examined one time each with an CV of 1.8 and 1.2%, respectively.

## 4 Discussion

### 4.1 Overview

The aim was to systematically review the scientific literature regarding the validity and reliability of different methods to assess ASR, i.e., methods to assess MAS and MSS. The high number of studies and methods included in this review emphasizes the popularity of both MAS and MSS in research and practice. As a combination of MAS and MSS, ASR has the advantage of normalizing absolute values for individualizing high-intensity training above MAS to take individual tolerances to high-intensity efforts into account [1, 4].

Regarding MAS, CPET on a treadmill is mostly used as a gold standard method (21 out of 28 studies; criterion validity), but with different protocols or definitions of MAS. The most studied methods could be assigned to CPET (n = 19) and time trials (n = 20), followed by incremental continuous field tests such as the UMTT (n = 12). The least studied methods were shuttle runs (n = 10). Intraday (n = 1) and interday reliability (n = 3) of methods to assess MAS were investigated equally but overall very rarely.

With respect to MSS, radar measurements (11 out of 28 studies; criterion validity) and distances between 20 and 40 m (23 out of 28 studies) are mostly used as gold standard methods for validity testing. Validity was assessed for GPS/LPS measurements most times (n = 18), followed by timing gates including different split distances (n = 8), and video analysis (n = 2). Radar and laser were (n = 2) validated against a linear motorized encoder. Intraday reliability (n = 22) was investigated remarkedly more frequently than interday reliability (n = 2).

### 4.2 Maximal aerobic speed

#### 4.2.1 Cardiopulmonary exercise testing

Although CPET on a treadmill is considered as the gold standard method to assess MAS, the testing protocols or definitions, i.e., the methods on how to retrieve MAS based on CPET, are yet to be clarified. The most used protocols are (square-wave) incremental protocols with and without resting in between the stages with different starting velocities, increments or duration of stages, or protocols based on individual performance, e.g., starting speed related to theoretical maximal heart rate [6, 7, 10, 12–14, 32, 34–37, 39, 42–46, 48–50, 52]. The definitions of MAS used in the studies were also different, e.g., the first speed when VO_2_max occurred with no further specification [6, 39, 45, 46, 48, 49, 52], calculated based on VO_2_ kinetics [7, 32, 34, 35, 43, 44, 50] the first speed of the 30-s interval of VO_2_max [7, 12, 13, 50], the speed at the onset of the plateau of VO_2_ [10, 42], or the final speed reached during CPET [14, 35–37]. Regarding validity of CPET on a treadmill to determine MAS, different incremental continuous protocols yielded similar results when the increments and stage durations were multiples of each other (e.g. 1 km/h increment and 2 min duration of stages versus 0.5 km/h increment and 1 min duration of stages) [6]. For the same increments, longer durations of stages lead to lower results of MAS (-2.1±0.5 –-2.0±1.45 km/h) [7, 50]. MAS shows significantly higher values when using square wave protocols (with resting periods) than when examined by incremental continuous protocols [7, 50]. Because different protocols used in the CPET yield different results in MAS, no conclusion can be drawn in consideration of the included studies. However, taken the definition of MAS into account, i.e., the first speed associated with VO_2_max, it is crucial for the subjects to actually reach VO_2_max during the treadmill test. That can be verified by the incidence of a VO_2_ plateau or other criteria measures such as blood lactate, respiratory exchange ratio, heart rate, or the rating of perceived exertion [81]. Regarding the protocol, continuous incremental exercises with durations between 8 and 16 min (e.g., starting at 4 or 6 km/h for recreational subjects and at 8 or 10 km/h for trained subjects with 1 km/h increments every minute) are suggested to reach VO_2_max and might therefore also be suitable for assessing MAS [81, 82]. Additionally, the duration of stages should potentially be chosen differently depending on the background of the athletes. For example, athletes accustomed to prolonged running (e.g., long distance runners) might benefit from longer stages (i.e., 2 or 3 min) whereas athletes accustomed to shorter or more intense running (e.g., some team sports athletes or 400-m runners) would suffer from neuromuscular exhaustion during longer stages and therefore benefit from shorter stages (i.e., 1 min). When MAS was determined at the onset of the plateau of VO_2_max, i.e., VO_2_ reaching a steady-state during an incremental treadmill test, the results differed remarkedly from MAS determined at the 30-s interval of VO_2_max [10]. These discrepancies might be explained by the definition of a leveling-off of VO_2_ lasting at least 1 min [83] and the 30-s interval of VO_2_max almost always occurs at the end of an incremental exercise close to the maximal speed [10, 11]. Further results regarding comparisons of definitions of MAS indicate that the final speed reached during incremental exercise as well as velocities based on calculations or extrapolations of VO_2_ to assess MAS are associated with a higher input of anaerobic resources than the first speed at the steady-state of VO_2_, i.e. the speed at the onset of the plateau of VO_2_max and therefore the first speed associated with VO_2_max when mainly aerobic resources are used [35, 39]. Such considerations should be taken into account when assessing MAS with CPET to determine the first speed associated with VO_2_max at which energy production is still largely aerobic [10, 11]. Especially for the purpose of HIIT prescription based on MAS or ASR, using definitions as the speed of the 30-s interval of VO_2_max or the final velocity reached during CPET instead of the velocity at the onset of the VO_2_ plateau could lead to different adaptations as intended or even overtraining.

The one study addressing interday reliability for MAS determined by CPET reported good relative reliability. However, since different protocols on the treadmill and different definitions of MAS are currently used and partly differ regarding their results, further studies should address validity and especially reliability of a consistent definition of MAS.

#### 4.2.2 Incremental continuous field tests

Léger et al. [84] developed and validated an incremental continuous field test to indirectly determine VO_2_max–the UMTT. Further, the UMTT and variations of it (e.g., VAM-EVAL) were then used to estimate MAS as they are less time and material consuming and need less expertise to implement than CPET [44, 85]. Regarding the validity of these field tests, the results depend on the protocols and definitions used in the reference measure and in the field test. In all of the studies (n = 8) investigating the validity of incremental continuous field tests to estimate MAS, CPET was used as the reference measure (criterion validity). When the identical protocols were implemented in the CPET and the field tests, two studies reported good validity [45, 48]. However, one study reported significant differences [52]. Lopes et al. [45] and Pallarés et al. [48] validated the UMTT, whereas Cappa et al. [52] developed a new field test (i.e., University of Catamarca test) and validated it with CPET on a treadmill with the same protocol. The University of Catamarca test consisted of a hexagon with 20-m long sides so that the participants ran around corners and not as in the UMTT on a linear and curved track potentially leading to an earlier exhaustion in the Catamarca test [52]. Comparisons of the final speed reached in the UMTT with the MAS calculated according to Lacour et al. [44] showed good validity [35, 44], whereby heterogenous results were found when the maximal speed during CPET [35, 36], or the speed associated with VO_2_max [6, 45, 48]were used as the definitions of MAS during CPET. The final velocity reached during the UMTT resulted in higher values (+1.61 km/h) than the speed at the onset of the VO_2_ plateau (as assessed with CPET) [10]. Therefore, using the final velocity of the UMTT to estimate MAS and prescribe training, e.g. HIIT, might lead to higher intensities as intended what can be fatal in terms of maladaptation or even overtraining. Although no study on reliability could be included, Léger et al. [84] reported good reliability of the maximal metabolic equivalents related to the UMTT.

#### 4.2.3 Time trials

Set-distance time trials with different distances or 5-min time trials are often used in research and practice to estimate MAS because of an easy implementation even for larger groups [3, 33]. While the average speed during 5-min time trials achieved similar results as the final speed reached during CPET or the UMTT in men of different fitness or amateur soccer players [36, 37, 40], the results on the validity of set-distance time trials are heterogeneous. When 1500 or 1610-m time trials were compared to CPET (criterion validity), all of the studies reported an overestimation of MAS investigating different participants, such as male and female runners, male Australian rules football players, or male trained soccer players, and with different definitions and protocols used in the CPET [10, 12, 43, 44, 46]. Bellenger et al. [33] and Lundquist et al. [47] investigated set-distance time trials between 1200 and 2200 m with male and female Australian rules football players. However, they used the final speed reached in the UMTT as the reference measure (convergent validity). For female subjects, the smallest effect and largest relationship was examined between the 1400-m time trial and the UMTT [47], whereby for male subjects the smallest effect was found for the 2000-m time trial [33]. These results indicate that the validity of set-distance time trials depends on which distance is used for which sex, type of sports, or fitness. For this reason, Bellenger et al. [33] suggested to predict MAS from the running speed for any time trial distance between 1200 and 2200 m with the equation: MAS = average speed * (0.766 + 0.117 * distance). However, the validity of this equation could not be confirmed for a 1500-m time trial in trained male soccer players [10]. When the studies investigating criterion validity of time trials with an accepted gold standard, i.e. CPET, are considered (n = 6), the results indicate that the time or distance should be selected according to the background of the subject (sex, type of sports, fitness). For lower fitness or subjects associated with sports based on higher speeds and mainly anaerobic energy supply, e.g. most team sport players or sprinters, shorter distances might be favorable (e.g., 1200–1400 m), whereas for (endurance) trained athletes longer distances (e.g., 1500–2000 m) could reflect an average speed more similar to MAS [10, 36, 37, 43, 44, 46]

#### 4.2.4 Shuttle runs

Shuttle runs are defined by stages with increasing speed or distance and changes of direction, often by 180°. Most shuttle-runs include active or passive rest [11]. While the peak speed of shuttle runs with passive rest (futsal specific shuttle run; 30–15 IFT) overestimated MAS assessed during CPET [14, 32], shuttle-runs with active or no rest (Carminatti’s test, 20-m shuttle run) yielded more similar results to CPET (criterion validity) [42, 49]. The passive rest could have provided an opportunity to recover in-between the shuttles so that a higher final speed was reached [11, 14]. The results for the 30–15 IFT and the 3-min 30-s endurance capacity test, showed good interday and intraday reliability, respectively [14, 53]. When only studies using an accepted gold standard to assess MAS, i.e. CPET (n = 4; criterion validity), are considered, most of the studies (two out of three) determining final speed during a shuttle run could not confirm its validity (large overestimation by shuttle runs) [14, 32]. These results might be mainly due to shuttle runs demanding more anaerobic resources and a higher energy cost of running because of change of direction movements [11]. These results indicate that MAS cannot be examined by the final speed reached during a shuttle run.

#### 4.2.5 Other/Individualized tests

Sangan et al. [13] validated a field test that is based on individual RPE during three stages (RPE 10, 13, and 17) lasting three minutes each. The comparisons of the average velocities during the last minutes of the three stages and MAS retrieved by CPET showed poor criterion validity, while absolute and relative intraday reliability can be rated as good. While validity for the three-level tests needs to be investigated further with CPET, validity of a field tests based on RPE cannot be confirmed [13, 51].

### 4.3 Maximal sprinting speed

#### 4.3.1 Radar/Laser technology

As the MSS reflects the maximal overall possible sprinting speed a subject can reach, it marks the upper limit of the ASR by providing information about the maximal physiological, mechanical, and coordinative output during sprinting [1]. Measuring the speed profile during linear sprinting with laser or radar devices is considered as the gold standard method to examine MSS [18, 76]. One study examined and confirmed convergent validity of both radar and laser measurement during 40-m sprints to assess MSS with a linear motorized encoder as the reference measure [70]. The validation of accepted gold standard methods becomes challenging in the absence of alternative gold standards. However, previous studies have demonstrated the accuracy of laser and radar devices in assessing various sprinting characteristics [21]. Both absolute and relative intraday reliability was reported good in all studies (n = 4) [17, 70, 71, 79] as well as absolute and relative interday reliability (n = 2) [17, 71].

#### 4.3.2 Timing gates

Timing gates are commonly used to assess sprinting characteristics as they conveniently provide sprinting times for distinct distances [27, 79]. When the average speed assessed during 5- and 10-m splits was compared to MSS measured with radar or a linear motorized encoder [62, 70, 79], the results indicate that 10-m splits are also sufficient to assess MSS with timing gates compared to 5-m splits. Moreover, Zabaloy et al. [79] did not detect significant differences in the 20–25 and 25–30-m splits indicating that participants reached MSS between 20 and 30 m. With respect to the total distances for the assessment of MSS, distances between 20 and 100 m are used in the included studies [60, 69]. Although no study compared different total distances, results of comparisons of the split times indicate, that distances between 20 and 40 m should cover an achievement of MSS for team sports athletes [57, 63, 64, 80]. Conversely, studies investigating trained track and field sprinters revealed an achievement of MSS between 40 and 70 m [86, 87]. Regarding the intraday reliability of timing gates during 30–50-m linear sprints, good absolute and relative reliability was reported [65, 70, 79, 80]. Although no studies on interday reliability of assessing MSS with timing gates could be included in this review, studies investigating other sprinting characteristics (e.g., acceleration) confirmed interday reliability of timing gates. Therefore, it can be assumed that interday reliability would be acceptable for MSS as well [27, 88]. In sum, timing gates with 5- or 10-m split times seem to be a valid and reliable method to assess MSS during linear sprints between 20 and 40 m for team sports with indications that elite sprinters might need longer distances to reach MSS [57, 62–65, 70, 79, 80, 86]. However, no results regarding total distances and split times for the examination of MSS with timing gates are available for recreational subjects who might need different distances due to the lack of expertise and lower fitness.

#### 4.3.3 Global and local positioning systems

GPS and LPS have recently become more accessible and affordable to assess running or sprinting characteristics, e.g. MSS. They allow receiving live speed and distance data from multiple subjects simultaneously during testing sessions but also during training or matches [59]. Regarding the validity of GPS measures during linear sprinting to assess MSS, some of the studies using radar as the reference method (n = 6) reported good criterion validity for 10 Hz GPS (three out of four) [18, 62, 77]. However, Akyildiz et al. [66] examined rather poor criterion validity for 10 Hz GPS with comparable samples (i.e., male amateur soccer players) and sprinting distances (i.e., 40 m). In addition, 5 Hz and 16 Hz GPS also yielded different results in MSS compared to radar (criterion validity) [15, 75]. 20–45 Hz LPS systems obtained rather poor validity when compared to motion capture [58, 61], indicating that LPS measures are not yet applicable for the assessment of MSS. These results seem surprising as LPS was considered a valid technology to determine other running characteristics, though Pino-Ortega et al. [89] point out that validity of LPS is highly dependent on the data processing. Most of the studies (seven out of 10) reported good intraday reliability for MSS assessed with 1–20 Hz GPS/LPS [67, 69, 70, 73, 74, 77, 78] and the one study that assessed interday reliability of 10 Hz GPS showed good absolute reliability [18]. To conclude, most studies using an accepted gold standard method to assess MSS, i.e. radar technology, could not confirm criterion validity of GPS or showed inadequate methodological quality (four out of six studies) [15, 62, 66, 75]. LPS measurements showed poor criterion and convergent validity in all studies relating to motion capture (n = 2) [58, 61]. Interday (one out of one study) and intraday reliability (seven out of 10 studies) was reported as good for GPS and LPS in most studies [18, 67, 69, 70, 73, 74, 77, 78].

#### 4.3.4 Video analysis *via* smartphone applications

A relatively cheap and easily accessible method to assess sprint kinematics is video analysis via smartphone applications that record time and distance data to calculate for example velocities. Two studies validated a smartphone app (MySprint App) with laser or radar measurements. Results regarding MSS showed good criterion validity in male and female team sport athletes and in male trained sprinters as well as good intraday reliability [71, 76]. The results of these two studies investigating a smartphone app indicate that this method can be used validly and reliably to assess MSS; however, with respect to the small number of studies, further investigations are crucial.

#### 4.3.5 Treadmill sprinting

Since MSS is usually measured during linear sprinting on a (outdoor) track, several environmental conditions can influence the outcome measures. Therefore, standardizing by implementing treadmills in the laboratory was previously suggested [72]. However, the studies examining (non-) motorized treadmills to assess MSS showed poor convergent and criterion validity (large ES) as running on a treadmill yielded much lower MSS than overground running [60, 72]. This difference is likely a result of the participants having to overcome the high inherent resistance of the treadmill. Though, intraday and interday reliability was reported as good [72].

### 4.4 Strengths and limitations

#### 4.4.1 Strengths

The results of this review should be interpreted concerning its limitations and strengths as well as the limitations and strengths of the included studies. A strength of this article is the large number of studies (n = 58) that was included in the analysis. In addition, the studies’ participants were associated to different backgrounds allowing conclusions for several fitness and settings for adults. A major methodological strength of this review is the application of the COSMIN checklist which is especially suited to evaluate the risk of bias of validity and reliability studies [29, 30].

#### 4.4.2 Limitations

A limitation is the unequal numbers of studies that could be included for the investigated methods, e.g. only few studies on shuttle runs to estimate MAS or on video analysis to assess MSS. This issue needs to be addressed in the future, so that clearer conclusions can be drawn. Studies in this article can be criticized since a substantial number of studies (50%) used not as gold standard accepted reference methods. Methodological quality of studies reporting validity for the assessment of MSS were mostly rated *inadequate* or *doubtful* (70%). For interday and intraday reliability, 50% of the studies were rated *very good* or *adequate*, and 50% were rated *doubtful* or *inadequate*. These findings can be explained by the inclusion criteria, as there was no restriction on the type of studies. Hence, studies in which validity or reliability assessment for MSS was not the main objective were included as well. Although they were well designed for their main purpose, the information needed for validity or reliability assessment was not always provided. Additionally, many studies reporting validity used very small sample sizes and hence were rated with a low methodological quality. Another limitation of the included studies is the fact that in studies on MAS as well as MSS mostly male participants were used and very few female participants. Moreover, in studies examining MSS mostly team sport athletes were included, potentially because MSS is rather crucial in team sports and sports teams are attractive samples as they usually consist of more than 20 players per team [21].

### 4.5 Practical applications & future research

When performance tests are implemented to monitor individual performance changes or to retrieve values for training prescription, these methods have to be highly valid and reliable [90], i.e., with respect to ASR, the methods to assess MAS and MSS. Practitioners and researchers should be aware that the results of methods for CPET to assess MAS indicate that the final speed reached during the test or the speed of the 30-s interval of VO_2_max are associated with a higher input of anaerobic energy supply than when the speed at the onset of the plateau of VO_2_ is determined. Therefore, the speed at the onset of this plateau seem to reflect true MAS and should be assessed [10, 91]. The results for time trials indicate that distances need to be selected based on the fitness, sex, or type of sports of the participants so that the results for MAS might be valid. We recommend that for subjects with a lower fitness, team sports athletes, or sprinters shorter distances should be used (e.g. 1200–1400 m), while longer distances are more favorable for endurance trained athletes to estimate MAS (e.g. 1500–2000 m) [10, 12, 33, 43, 47]. In addition, the results for continuous incremental field tests or shuttle runs independently of subjects’ fitness [10, 14, 32, 45, 48, 52] point out that these tests are not suitable to estimate true MAS.

Besides using radar or laser measurement during linear sprinting [17, 70, 79] timing gates with split times of 5 or 10 m and video analysis with a smartphone application seem promising when determining MSS [62, 71, 76, 79]. GPS measurements during linear sprinting showed poor validity and the maximal velocities measured during training and matches [15, 19, 59, 62, 66] as well as the maximal speed achieved during treadmill sprinting could not reach the actual MSS [15, 19, 59, 60, 62, 66, 72]. However, to delineate between type of sports, sexes, or fitness, future studies should address population specific validity. From a practical perspective, shorter total distances (20–40 m) are suggested for recreational athletes or type of sports in which shorter sprints are common, e.g. team sports such as soccer or handball, whereas trained sprinters might need distances between 40 and 70 m to reach MSS [57, 62–65, 70, 79, 80, 86]. It is noteworthy that several influencing factors such as wind, temperature, running surface, or shoes can affect the sprint performance and thus potentially MSS. These factors should therefore always be considered when determining sprint performance, especially when assessing changes in intraindividual performance over time [21].

**Fig 2:**
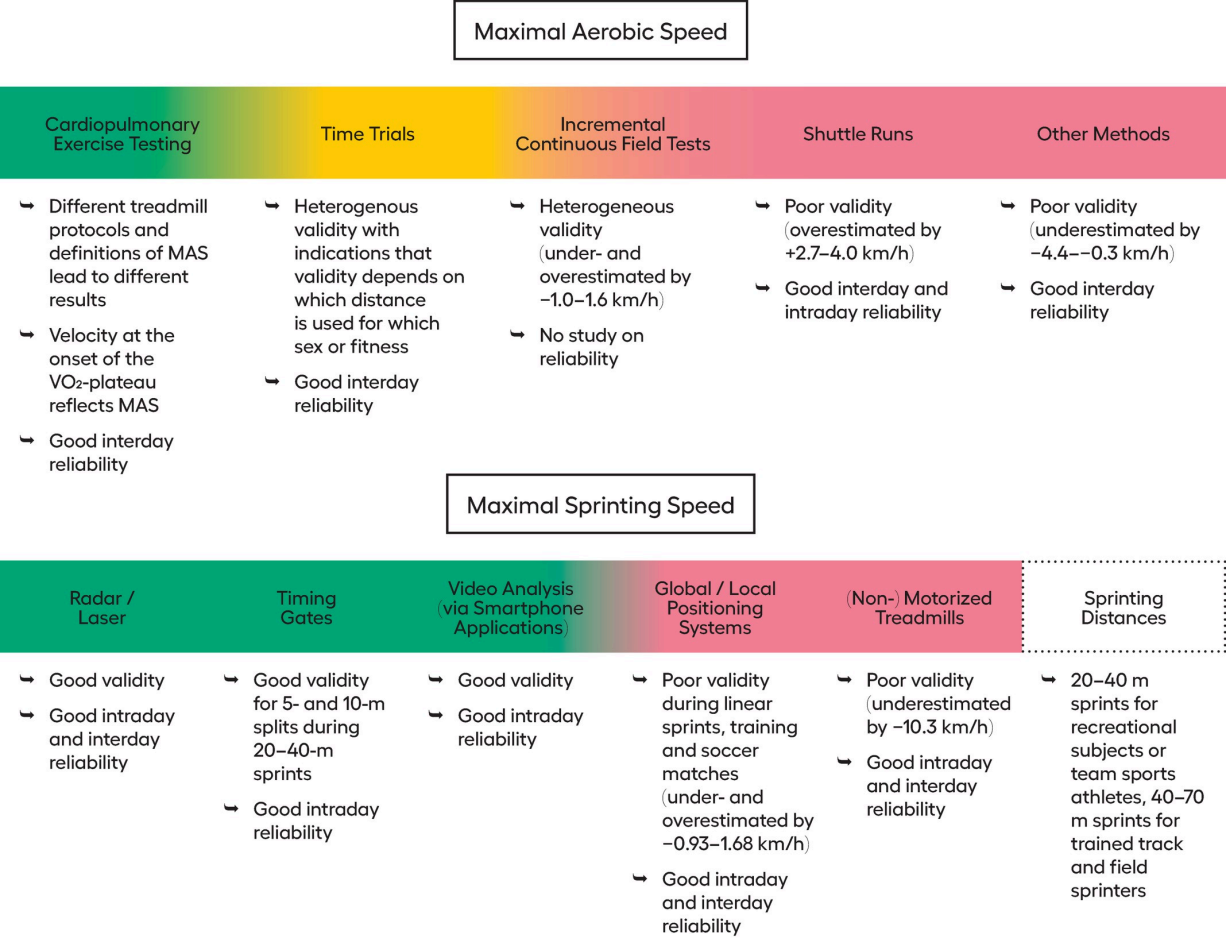
Overview of conclusions for the testing methods for maximal aerobic speed and maximal sprinting speed. The color grading reflects the rating for criterion validity—from green indicating good validity to red indicating poor validity. *VO2* Oxygen Uptake, *MAS* Maximal Aerobic Speed.

Both the selection of methods and the evaluation of results for ASR should be based on the sport-specific requirements. While different team sports such as soccer or volleyball have different game demands (i.e. shorter sprints and lower total distance covered during a volleyball match compared to soccer), the testing methods might also differ, e.g. lower time trial distances to assess MAS and lower sprinting distances to assess MSS for volleyball players compared to soccer players. However, future research might focus on these aspects. An overview of the conclusions and practical applications is presented in Fig 2.

**Fig 3:**
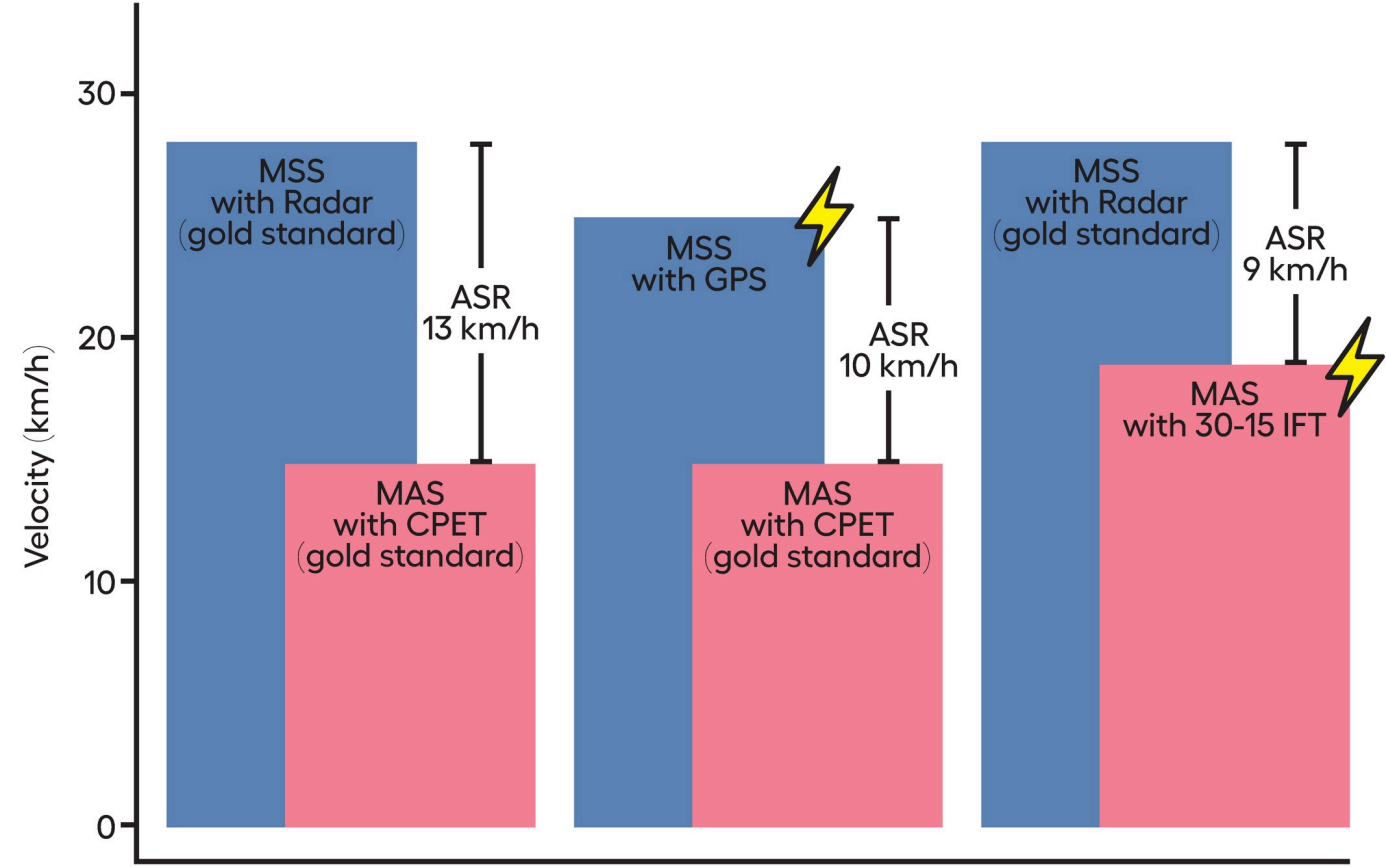
Illustration of values for MAS, MAS and ASR when using different testing methods. Presented values are based on the results of Čović et al. [14] for the MAS data and Djaoui et al. [19] for the MSS data. When GPS is used, the MSS will most likely be underestimated compared to radar (approximately 3 km/h) (17). MAS will most likely be overestimated when the 30–15 IFT is implemented compared to CPET on a treadmill (approximately 4 km/h) [12]. The ASR will change accordingly. *MAS* Maximal Aerobic Speed, *MSS* Maximal Sprinting Speed, *ASR* Anaerobic Speed Reserve, *CPET* Cardiopulmonary Exercise Testing, *GPS* Global Positioning System, *30–15 IFT* 30–15 Intermittent Fitness Test.

With respect to controlling training interventions, an example of high-intensity-interval prescriptions with ASR might illustrate the issue of using different methods when assessing the control parameters. When MAS and MSS are retrieved by CPET and radar measurement, respectively, a recreational runner might achieve results of 15 km/h for MAS and 28 km/h for MSS and accordingly an ASR of 13 km/h. However, when for instance the 30–15 IFT is used instead of CPET to set the lower boundary of ASR, the estimated MAS might yield 19 km/h what reduces ASR to 9 km/h [14]. The prescription of high-intensity-intervals with the intensity of for example 30% ASR (MAS + 30% ASR) will therefore be noticeably more intense with MAS retrieved by the 30–15 IFT than with CPET (i.e., 18.9 km/h for true MAS and 21.7 km/h for 30–15 IFT). Conversely, the intensity would be too low (approx. 18.2 km/h) for the athlete when MSS is assessed for example during matches or training using GPS [19] (see Fig 3). Though, only few studies exist on training interventions using ASR [1, 92–94], further research is needed to fill this gap.

### 4.6 Conclusions

Beyond assessing single performance parameters, the ASR can provide further insights into an athlete’s physiological and neuromuscular profile by considering the individual tolerance to high-intensity exercise. As ASR consists of the parameters MAS and MSS, the methods to assess these parameters need to be valid and reliable.

MAS can be defined during CPET, yet there are different testing protocols without a consensus about the most appropriate one. Due to physiological considerations based on energy supply, the speed at the onset of the VO_2_-plateau seems to be the most appropriate method to determine true MAS. For field tests, studies’ results on validity are heterogeneous and do not favor a specific field test (incremental continuous or shuttle runs) for the determination of MAS. However, results on time trials indicate that distances adapted to the subjects’ sporting background, fitness or sex might be suitable to estimate MAS.

Regarding MSS, linear sprints using timing gates or video analysis seem to provide valid and reliable results besides the gold standard method, i.e. radar or laser measurements. The validity of GPS or sprinting on a treadmill cannot be confirmed. Sprinting distances between 20 and 40 m should be selected for recreational subjects or type of sports in which shorter sprints are crucial, e.g. team sports, whereas trained track and field sprinters might need longer total distances (40 to 70 m) to reach MSS.

In particular the use for prescribing training emphasizes the importance of valid and reliable measurements of MAS and MSS to achieve optimal and desired intensity based on ASR. Methods–ideally with a low measurement error and therefore a high reliability–should be maintained throughout the training routine so that changes in ASR can be attributed to changes in the individual performance and not to differing results because of the testing method.

## Supporting information

S1 Table PRISMA 2020 checklist. (DOCX)

S2 Table Assessment of methodological quality based on the boxes 6 – 9a of the COSMIN checklist [29, 30]. (DOCX)

S1 File Search terms PubMed. (DOCX)
