# Supplementary material for: Assessing anaerobic speed reserve: A systematic review on the validity and reliability of methods to determine maximal aerobic speed and maximal sprinting speed in running-based sports
Source: PLoS One. 2024 Jan 22;19(1):e0296866. doi: 10.1371/journal.pone.0296866 (PMC10802961; doi:10.1371/journal.pone.0296866)
Supplement: S2 Table — (DOCX) [file pone.0296866.s002.docx]

**Supplementary Information 4** Assessment of methodological quality based on the boxes 6 – 9a of the COSMIN checklist [29, 30]

|  | Akyildiz et al. [66] | Barbero-Alvarez et al. [32] | Barbero-Alvarez et al. [55] | Beato et al. [18] | Beato et al. [67] | Bellenger et al. [33] | Benhammou et al. [51] | Bernard et al. [34] | Berthoin et al. [35] |  |  |  |  |
| --- | --- | --- | --- | --- | --- | --- | --- | --- | --- | --- | --- | --- | --- |
| **Box 6. Reliability** | | | |  |  |  |  |  |  |  |  |  |  |
| Design requirements | | | |  |  |  |  |  |  |  |  |  |  |
| 1. Were patients stable in the interim period on the construct to be measured? | 3 | NA | NA | 3 | 1 | NA | 3 | NA | NA |  |  |  |  |
| 2. Was the time interval appropriate? | 3 | NA | NA | 3 | 1 | NA | 3 | NA | NA |  |  |  |  |
| 3. Were the test conditions similar for the measurements? e.g. type of administration, environment, instructions | 3 | NA | NA | 3 | 3 | NA | 3 | NA | NA |  |  |  |  |
| Statistical methods | | | |  |  |  |  |  |  |  |  |  |  |
| 4. For continuous scores: Was an intraclass correlation coefficient (ICC) calculated? | 3 | NA | NA | 1 | 3 | NA | 3 | NA | NA |  |  |  |  |
| Other | | | |  |  |  |  |  |  |  |  |  |  |
| 8. Were there any other important flaws in the design or statistical methods of the study? | 3 | NA | NA | 3 | 3 | NA | 3 | NA | NA |  |  |  |  |
| **Box 7. Measurement error** |  |  |  |  |  |  |  |  |  |  |  |  |  |
| Design requirements | | | |  |  |  |  |  |  |  |  |  |  |
| 1. Were patients stable in the interim period on the construct to be measured? | 3 | NA | NA | 3 | 1 | NA | 3 | NA | NA |  |  |  |  |
| 2. Was the time interval appropriate? | 3 | NA | NA | 3 | 1 | NA | 3 | NA | NA |  |  |  |  |
| 3. Were the test conditions similar for the measurements? (e.g. type of administration, environment, instructions) | 3 | NA | NA | 3 | 3 | NA | 3 | NA | NA |  |  |  |  |
| Statistical methods | | | |  |  |  |  |  |  |  |  |  |  |
| 4. For continuous scores: Was the Standard Error of Measurement (SEM), Smallest Detectable Change (SDC) or Limits of Agreement (LoA) calculated? | 3 | NA | NA | 2 | 3 | NA | 3 | NA | NA |  |  |  |  |
| Other | | | |  |  |  |  |  |  |  |  |  |  |
| 6. Were there any other important flaws in the design or statistical methods of the study? | 3 | NA | NA | 3 | 3 | NA | 3 | NA | NA |  |  |  |  |
| **Box 8. Criterion validity** | | | |  |  |  |  |  |  |  |  |  |  |
| Statistical methods | | | |  |  |  |  |  |  |  |  |  |  |
| 1. For continuous scores: Were correlations, or the area under the receiver operating curve calculated? | 3 | 3 | 3 | 3 | 3 | 3 | 3 | 3 | 3 |  |  |  |  |
| Other | | | |  |  |  |  |  |  |  |  |  |  |
| 3. Were there any other important flaws in the design or statistical methods of the study? | 3 | 1 | 1 | 3 | 3 | 3 | 3 | 1 | 1 |  |  |  |  |
| **Box 9a. Convergent validity** | | | |  |  |  |  |  |  |  |  |  |  |
| Design requirements | | | |  |  |  |  |  |  |  |  |  |  |
| 1 Is it clear what the comparator instrument(s) measure(s)? | 3 | 3 | 2 | 3 | 3 | 3 | 3 | 3 | 3 |  |  |  |  |
| 2 Were the measurement properties of the comparator instrument(s) sufficient? | 3 | 3 | 2 | 3 | 3 | 3 | 3 | 3 | 3 |  |  |  |  |
| Statistical methods | | | |  |  |  |  |  |  |  |  |  |  |
| 3 Were design and statistical methods adequate for the hypotheses to be tested? | 3 | 3 | 3 | 3 | 3 | 3 | 3 | 3 | 3 |  |  |  |  |

3 = very good; 2 = adequate; 1 = doubtful; 0 = inadequate, and NA = not applicable.

**Supplementary Information 4** Continued.

|  | Berthon et al. [36] | Berthon et al. [37] | Billat et al. [6] | Cappa et al. [52] | Carminatti et al. [38] | Chahal et al. [68] | Ghigiarelli et al. [71] | Clark et al. [56] | Coutts and Duffield [69] | Čović et al. [14] | Da Silva and Machado [39] |  |  |  |
| --- | --- | --- | --- | --- | --- | --- | --- | --- | --- | --- | --- | --- | --- | --- |
| **Box 6. Reliability** | | | |  |  |  |  |  |  |  |  |  |  |  |
| Design requirements | | | |  |  |  |  |  |  |  |  |  |  |  |
| 1. Were patients stable in the interim period on the construct to be measured? | NA | NA | NA | 3 | NA | 3 | 3 | NA | 3 | 3 | NA |  |  |  |
| 2. Was the time interval appropriate? | NA | NA | NA | 1 | NA | 3 | 3 | NA | 3 | 3 | NA |  |  |  |
| 3. Were the test conditions similar for the measurements? e.g. type of administration, environment, instructions | NA | NA | NA | 3 | NA | 3 | 3 | NA | 3 | 3 | NA |  |  |  |
| Statistical methods | | | |  |  |  |  |  |  |  |  |  |  |  |
| 4. For continuous scores: Was an intraclass correlation coefficient (ICC) calculated? | NA | NA | NA | 1 | NA | 3 | 3 | NA | 0 | 3 | NA |  |  |  |
| Other | | | |  |  |  |  |  |  |  |  |  |  |  |
| 8. Were there any other important flaws in the design or statistical methods of the study? | NA | NA | NA | 3 | NA | 3 | 3 | NA | 1 | 3 | NA |  |  |  |
| **Box 7. Measurement error** |  |  |  |  |  |  |  |  |  |  |  |  |  |  |
| Design requirements | | | |  |  |  |  |  |  |  |  |  |  |  |
| 1. Were patients stable in the interim period on the construct to be measured? | NA | NA | NA | 3 | NA | 3 | 3 | NA | 3 | 3 | NA |  |  |  |
| 2. Was the time interval appropriate? | NA | NA | NA | 1 | NA | 3 | 3 | NA | 3 | 3 | NA |  |  |  |
| 3. Were the test conditions similar for the measurements? (e.g. type of administration, environment, instructions) | NA | NA | NA | 3 | NA | 3 | 3 | NA | 3 | 3 | NA |  |  |  |
| Statistical methods | | | |  |  |  |  |  |  |  |  |  |  |  |
| 4. For continuous scores: Was the Standard Error of Measurement (SEM), Smallest Detectable Change (SDC) or Limits of Agreement (LoA) calculated? | NA | NA | NA | 2 | NA | 3 | 3 | NA | 0 | 3 | NA |  |  |  |
| Other | | | |  |  |  |  |  |  |  |  |  |  |  |
| 6. Were there any other important flaws in the design or statistical methods of the study? | NA | NA | NA | 3 | NA | 3 | 3 | NA | 1 | 3 | NA |  |  |  |
| **Box 8. Criterion validity** | | | |  |  |  |  |  |  |  |  |  |  |  |
| Statistical methods | | | |  |  |  |  |  |  |  |  |  |  |  |
| 1. For continuous scores: Were correlations, or the area under the receiver operating curve calculated? | 3 | 3 | 3 | 3 | 3 | 0 | 3 | 3 | 3 | 3 | 3 |  |  |  |
| Other | | | |  |  |  |  |  |  |  |  |  |  |  |
| 3. Were there any other important flaws in the design or statistical methods of the study? | 3 | 3 | 3 | 3 | 3 | 3 | 3 | 1 | 0 | 3 | 3 |  |  |  |
| **Box 9a. Convergent validity** | | | |  |  |  |  |  |  |  |  |  |  |  |
| Design requirements | | | |  |  |  |  |  |  |  |  |  |  |  |
| 1 Is it clear what the comparator instrument(s) measure(s)? | 3 | 3 | 3 | 3 | 3 | 3 | 3 | 3 | 3 | 3 | 3 |  |  |  |
| 2 Were the measurement properties of the comparator instrument(s) sufficient? | 3 | 3 | 3 | 3 | 3 | 2 | 3 | 3 | 3 | 3 | 3 |  |  |  |
| Statistical methods | | | |  |  |  |  |  |  |  |  |  |  |  |
| 3 Were design and statistical methods adequate for the hypotheses to be tested? | 3 | 3 | 3 | 3 | 3 | 3 | 3 | 3 | 1 | 3 | 3 |  |  |  |

3 = very good; 2 = adequate; 1 = doubtful; 0 = inadequate, and NA = not applicable.

**Supplementary Information 4** Continued.

|  | Darendeli et al. [40] | Dillon et al. [41] | Dittrich et al. [42] | Djaoui et al. [19] | Ferro et al. [57] | Fleureau et al. [58] | Fornasier-Santos et al. [70] | Foster et al. [43] | Helland et al. [65] | Highton et al. [72] | Hoppe et al. [73] |  |  |  |
| --- | --- | --- | --- | --- | --- | --- | --- | --- | --- | --- | --- | --- | --- | --- |
| ***Box 6. Reliability*** | | | | |  |  |  |  |  |  |  |  |  |  |
| Design requirements | | | | |  |  |  |  |  |  |  |  |  |  |
| 1. Were patients stable in the interim period on the construct to be measured? | NA | NA | NA | NA | NA | NA | 3 | NA | 3 | 3 | 3 |  |  |  |
| 2. Was the time interval appropriate? | NA | NA | NA | NA | NA | NA | 3 | NA | 3 | 3 | 3 |  |  |  |
| 3. Were the test conditions similar for the measurements? e.g. type of administration, environment, instructions | NA | NA | NA | NA | NA | NA | 3 | NA | 3 | 3 | 3 |  |  |  |
| *Statistical methods* | | | | |  |  |  |  |  |  |  |  |  |  |
| 4. For continuous scores: Was an intraclass correlation coefficient (ICC) calculated? | NA | NA | NA | NA | NA | NA | 0 | NA | 1 | 0 | 0 |  |  |  |
| Other | | | | |  |  |  |  |  |  |  |  |  |  |
| 8. Were there any other important flaws in the design or statistical methods of the study? | NA | NA | NA | NA | NA | NA | 3 | NA | 3 | 3 | 3 |  |  |  |
| **Box 7. Measurement error** |  |  |  |  |  |  |  |  |  |  |  |  |  |  |
| Design requirements | | | | |  |  |  |  |  |  |  |  |  |  |
| 1. Were patients stable in the interim period on the construct to be measured? | NA | NA | NA | NA | NA | NA | 3 | NA | 3 | 3 | 3 |  |  |  |
| 2. Was the time interval appropriate? | NA | NA | NA | NA | NA | NA | 3 | NA | 3 | 3 | 3 |  |  |  |
| 3. Were the test conditions similar for the measurements? (e.g. type of administration, environment, instructions) | NA | NA | NA | NA | NA | NA | 3 | NA | 3 | 3 | 3 |  |  |  |
| Statistical methods | | | | |  |  |  |  |  |  |  |  |  |  |
| 4. For continuous scores: Was the Standard Error of Measurement (SEM), Smallest Detectable Change (SDC) or Limits of Agreement (LoA) calculated? | NA | NA | NA | NA | NA | NA | 3 | NA | 2 | 3 | 2 |  |  |  |
| Other | | | | |  |  |  |  |  |  |  |  |  |  |
| 6. Were there any other important flaws in the design or statistical methods of the study? | NA | NA | NA | NA | NA | NA | 3 | NA | 3 | 3 | 3 |  |  |  |
| **Box 8. Criterion validity** | | | | |  |  |  |  |  |  |  |  |  |  |
| Statistical methods | | | | |  |  |  |  |  |  |  |  |  |  |
| 1. For continuous scores: Were correlations, or the area under the receiver operating curve calculated? | 3 | 3 | 3 | 0 | 0 | 3 | 0 | 3 | NA | 3 | 0 |  |  |  |
| Other | | | | |  |  |  |  |  |  |  |  |  |  |
| 3. Were there any other important flaws in the design or statistical methods of the study? | 3 | 3 | 3 | 3 | 3 | 0 | 3 | 3 | NA | 3 | 3 |  |  |  |
| **Box 9a. Convergent validity** | | | | |  |  |  |  |  |  |  |  |  |  |
| Design requirements | | | | |  |  |  |  |  |  |  |  |  |  |
| 1 Is it clear what the comparator instrument(s) measure(s)? | 3 | 3 | 3 | 3 | 3 | 3 | 3 | 3 | NA | 3 | 3 |  |  |  |
| 2 Were the measurement properties of the comparator instrument(s) sufficient? | 3 | 3 | 3 | 3 | 3 | 3 | 3 | 3 | NA | 3 | 1 |  |  |  |
| Statistical methods | | | | |  |  |  |  |  |  |  |  |  |  |
| 3 Were design and statistical methods adequate for the hypotheses to be tested? | 3 | 3 | 3 | 3 | 3 | 1 | 3 | 3 | NA | 3 | 3 |  |  |  |

3 = very good; 2 = adequate; 1 = doubtful; 0 = inadequate, and NA = not applicable.

**Supplementary Information 4** Continued.

|  | Johnston et al. [74] | Johnston et al. [75] | Lacome et al. [15] | Lacour et al. [44] | Laursen et al. [54] | Lopes et al. [45] | Lorenzen et al. [46] | Lundquist et al. [47] | Massard et al. [59] | Morin and Sève [60] | Ogris et al. [61] |  |  |  |
| --- | --- | --- | --- | --- | --- | --- | --- | --- | --- | --- | --- | --- | --- | --- |
| **Box 6. Reliability** | | | |  |  |  |  |  |  |  |  |  |  |  |
| Design requirements | | | |  |  |  |  |  |  |  |  |  |  |  |
| 1. Were patients stable in the interim period on the construct to be measured? | 3 | 3 | NA | NA | 3 | NA | NA | NA | NA | NA | NA |  |  |  |
| 2. Was the time interval appropriate? | 3 | 3 | NA | NA | 3 | NA | NA | NA | NA | NA | NA |  |  |  |
| 3. Were the test conditions similar for the measurements? e.g. type of administration, environment, instructions | 3 | 3 | NA | NA | 3 | NA | NA | NA | NA | NA | NA |  |  |  |
| Statistical methods | | | |  |  |  |  |  |  |  |  |  |  |  |
| 4. For continuous scores: Was an intraclass correlation coefficient (ICC) calculated? | 3 | 3 | NA | NA | 3 | NA | NA | NA | NA | NA | NA |  |  |  |
| Other | | | |  |  |  |  |  |  |  |  |  |  |  |
| 8. Were there any other important flaws in the design or statistical methods of the study? | 3 | 3 | NA | NA | 3 | NA | NA | NA | NA | NA | NA |  |  |  |
| **Box 7. Measurement error** |  |  |  |  |  |  |  |  |  |  |  |  |  |  |
| Design requirements | | | |  |  |  |  |  |  |  |  |  |  |  |
| 1. Were patients stable in the interim period on the construct to be measured? | 3 | 3 | NA | NA | 3 | NA | NA | NA | NA | NA | NA |  |  |  |
| 2. Was the time interval appropriate? | 3 | 3 | NA | NA | 3 | NA | NA | NA | NA | NA | NA |  |  |  |
| 3. Were the test conditions similar for the measurements? (e.g. type of administration, environment, instructions) | 3 | 3 | NA | NA | 3 | NA | NA | NA | NA | NA | NA |  |  |  |
| Statistical methods | | | |  |  |  |  |  |  |  |  |  |  |  |
| 4. For continuous scores: Was the Standard Error of Measurement (SEM), Smallest Detectable Change (SDC) or Limits of Agreement (LoA) calculated? | 3 | 3 | NA | NA | 3 | NA | NA | NA | NA | NA | NA |  |  |  |
| Other | | | |  |  |  |  |  |  |  |  |  |  |  |
| 6. Were there any other important flaws in the design or statistical methods of the study? | 3 | 3 | NA | NA |  | NA | NA | NA | NA | NA | NA |  |  |  |
| **Box 8. Criterion validity** | | | |  |  |  |  |  |  |  |  |  |  |  |
| Statistical methods | | | |  |  |  |  |  |  |  |  |  |  |  |
| 1. For continuous scores: Were correlations, or the area under the receiver operating curve calculated? | 3 | 3 | 0 | 3 | NA | 3 | 0 | 3 | 0 | 3 | 0 |  |  |  |
| Other | | | |  |  |  |  |  |  |  |  |  |  |  |
| 3. Were there any other important flaws in the design or statistical methods of the study? | 0 | 0 | 0 | 3 | NA | 3 | 3 | 3 | 3 | 1 | 1 |  |  |  |
| **Box 9a. Convergent validity** | | | |  |  |  |  |  |  |  |  |  |  |  |
| Design requirements | | | |  |  |  |  |  |  |  |  |  |  |  |
| 1 Is it clear what the comparator instrument(s) measure(s)? | 3 | 3 | 3 | 3 | NA | 3 | 3 | 3 | 3 | 3 | 3 |  |  |  |
| 2 Were the measurement properties of the comparator instrument(s) sufficient? | 3 | 3 | 3 | 3 |  | 3 | 3 | 3 | 3 | 3 | 3 |  |  |  |
| Statistical methods | | | |  |  |  |  |  |  |  |  |  |  |  |
| 3 Were design and statistical methods adequate for the hypotheses to be tested? | 0 | 0 | 0 | 3 | NA | 3 | 3 | 3 | 3 | 3 | 1 |  |  |  |

3 = very good; 2 = adequate; 1 = doubtful; 0 = inadequate, and NA = not applicable.

**Supplementary Information 4** Continued.

|  | | Pallarés et al. [48] | Paradisis et al. [49] | Riboli et al. [7] | Riboli et al. [50] | Roe et al. [62] | Romero-Franco et al. [76] | Sagiroglu et al. [77] | Sandford et al. [12] | Sangan et al. [13] | Schnitzler et al. [53] | Simperingham et al. [17] |  |  |  |
| --- | --- | --- | --- | --- | --- | --- | --- | --- | --- | --- | --- | --- | --- | --- | --- |
|  | ***Box 6. Reliability*** | | | | |  |  |  |  |  |  |  |  |  |  |
|  | *Design requirements* | | | | |  |  |  |  |  |  |  |  |  |  |
| 1. Were patients stable in the interim period on the construct to be measured? | | NA | NA | NA | NA | NA | 3 | 3 | NA | 3 | 3 | 3 |  |  |  |
| 2. Was the time interval appropriate? | | NA | NA | NA | NA | NA | 3 | 3 | NA | 3 | 3 | 3 |  |  |  |
| 3. Were the test conditions similar for the measurements? e.g. type of administration, environment, instructions | | NA | NA | NA | NA | NA | 3 | 3 | NA | 3 | 3 | 3 |  |  |  |
|  | Statistical methods | | | | |  |  |  |  |  |  |  |  |  |  |
| 4. For continuous scores: Was an intraclass correlation coefficient (ICC) calculated? | | NA | NA | NA | NA | NA | 0 | 0 | NA | 3 | 3 | 3 |  |  |  |
|  | Other | | | | |  |  |  |  |  |  |  |  |  |  |
| 8. Were there any other important flaws in the design or statistical methods of the study? | | NA | NA | NA | NA | NA | 1 | 3 | NA | 3 | 3 | 3 |  |  |  |
| **Box 7. Measurement error** | |  |  |  |  |  |  |  |  |  |  |  |  |  |  |
|  | Design requirements | | | | |  |  |  |  |  |  |  |  |  |  |
| 1. Were patients stable in the interim period on the construct to be measured? | | NA | NA | NA | NA | NA | 3 | 3 | NA | 3 | 3 | 3 |  |  |  |
| 2. Was the time interval appropriate? | | NA | NA | NA | NA | NA | 3 | 3 | NA | 3 | 3 | 3 |  |  |  |
| 3. Were the test conditions similar for the measurements? (e.g. type of administration, environment, instructions) | | NA | NA | NA | NA | NA | 3 | 3 | NA | 3 | 3 | 3 |  |  |  |
|  | Statistical methods | | | | |  |  |  |  |  |  |  |  |  |  |
| 4. For continuous scores: Was the Standard Error of Measurement (SEM), Smallest Detectable Change (SDC) or Limits of Agreement (LoA) calculated? | | NA | NA | NA | NA | NA | 2 | 3 | NA | 2 | 2 | 3 |  |  |  |
|  | Other | | | | |  |  |  |  |  |  |  |  |  |  |
| 6. Were there any other important flaws in the design or statistical methods of the study? | | NA | NA | NA | NA | NA | 1 | 3 | NA | 3 | 3 | 3 |  |  |  |
|  | **Box 8. Criterion validity** | | | | |  |  |  |  |  |  |  |  |  |  |
|  | Statistical methods | | | | |  |  |  |  |  |  |  |  |  |  |
| 1. For continuous scores: Were correlations, or the area under the receiver operating curve calculated? | | 3 | 3 | 3 | 3 | 3 | 3 | 3 | 3 | 3 | 3 | NA |  |  |  |
|  | Other | | | | |  |  |  |  |  |  |  |  |  |  |
| 3. Were there any other important flaws in the design or statistical methods of the study? | | 3 | 3 | 3 | 3 | 0 | 1 | 3 | 1 | 3 | 3 | NA |  |  |  |
|  | **Box 9a. Convergent validity** | | | | |  |  |  |  |  |  |  |  |  |  |
|  | Design requirements | | | | |  |  |  |  |  |  |  |  |  |  |
| 1 Is it clear what the comparator instrument(s) measure(s)? | | 3 | 3 | 3 | 3 | 3 | 3 | 3 | 3 | 3 | 3 | NA |  |  |  |
| 2 Were the measurement properties of the comparator instrument(s) sufficient? | | 3 | 3 | 3 | 3 | 3 | 3 | 3 | 3 | 3 | 3 | NA |  |  |  |
|  | Statistical methods | | | | |  |  |  |  |  |  |  |  |  |  |
| 3 Were design and statistical methods adequate for the hypotheses to be tested? | | 3 | 3 | 3 | 3 | 1 | 1 | 3 | 1 | 3 | 3 | NA |  |  |  |

3 = very good; 2 = adequate; 1 = doubtful; 0 = inadequate, and NA = not applicable.

**Supplementary Information 4** Continued.

|  | Thron et al. [10] | Vescovi [62] | Willmott et al. [78] | Young et al. [64] | Zabaloy et al. [79] | Zabaloy et al. [80] |  |  |  |
| --- | --- | --- | --- | --- | --- | --- | --- | --- | --- |
| ***Box 6. Reliability*** | | | |  |  |  |  |  |  |
| *Design requirements* | | | |  |  |  |  |  |  |
| 1. Were patients stable in the interim period on the construct to be measured? | NA | NA | 3 | NA | 3 | 3 |  |  |  |
| 2. Was the time interval appropriate? | NA | NA | 3 | NA | 3 | 3 |  |  |  |
| 3. Were the test conditions similar for the measurements? e.g. type of administration, environment, instructions | NA | NA | 3 | NA | 3 | 3 |  |  |  |
| Statistical methods | | | |  |  |  |  |  |  |
| 4. For continuous scores: Was an intraclass correlation coefficient (ICC) calculated? | NA | NA | 3 | NA | 3 | 3 |  |  |  |
| Other | | | |  |  |  |  |  |  |
| 8. Were there any other important flaws in the design or statistical methods of the study? | NA | NA | 3 | NA | 3 | 3 |  |  |  |
| **Box 7. Measurement error** |  |  |  |  |  |  |  |  |  |
| Design requirements | | | |  |  |  |  |  |  |
| 1. Were patients stable in the interim period on the construct to be measured? | NA | NA | 3 | NA | 3 | 3 |  |  |  |
| 2. Was the time interval appropriate? | NA | NA | 3 | NA | 3 | 3 |  |  |  |
| 3. Were the test conditions similar for the measurements? (e.g. type of administration, environment, instructions) | NA | NA | 3 | NA | 3 | 3 |  |  |  |
| Statistical methods | | | |  |  |  |  |  |  |
| 4. For continuous scores: Was the Standard Error of Measurement (SEM), Smallest Detectable Change (SDC) or Limits of Agreement (LoA) calculated? | NA | NA | 3 | NA | 3 | 2 |  |  |  |
| Other | | | |  |  |  |  |  |  |
| 6. Were there any other important flaws in the design or statistical methods of the study? | NA | NA | 3 | NA | 3 | 3 |  |  |  |
| **Box 8. Criterion validity** | | | |  |  |  |  |  |  |
| Statistical methods | | | |  |  |  |  |  |  |
| 1. For continuous scores: Were correlations, or the area under the receiver operating curve calculated? | 3 | 0 | 3 | 3 | 3 | 0 |  |  |  |
| Other | | | |  |  |  |  |  |  |
| 3. Were there any other important flaws in the design or statistical methods of the study? | 1 | 3 | 1 | 3 | 3 | 3 |  |  |  |
| **Box 9a. Convergent validity** | | | |  |  |  |  |  |  |
| Design requirements | | | |  |  |  |  |  |  |
| 1 Is it clear what the comparator instrument(s) measure(s)? | 3 | 3 | 3 | 3 | 3 | 3 |  |  |  |
| 2 Were the measurement properties of the comparator instrument(s) sufficient? | 3 | 3 | 3 | 3 | 3 | 3 |  |  |  |
| Statistical methods | | | |  |  |  |  |  |  |
| 3 Were design and statistical methods adequate for the hypotheses to be tested? | 3 | 3 | 3 | 3 | 3 | 3 |  |  |  |

3 = very good; 2 = adequate; 1 = doubtful; 0 = inadequate, and NA = not applicable.
