# Supplementary material for: Assessing anaerobic speed reserve: A systematic review on the validity and reliability of methods to determine maximal aerobic speed and maximal sprinting speed in running-based sports
Source: PLoS One. 2024 Jan 22;19(1):e0296866. doi: 10.1371/journal.pone.0296866 (PMC10802961; doi:10.1371/journal.pone.0296866)
Supplement: S1 File — (DOCX) [file pone.0296866.s003.docx]

**Supplementary Information 3** Search Terms PubMed

(validity OR logical OR criterion OR convergent OR discrimination OR construct OR "gold standard" OR level OR standard OR reliability OR repeatability OR reproducibility OR "measurement error" OR consistency OR "smallest worthwhile change" OR "minimal detectable change", OR "typical error" OR usefulness OR „minimal important difference“ OR „standard error“ OR relationship OR relation OR association OR correlation)

AND

(„max* aerobic speed" OR MAS OR “maximal aerobic velocity” OR „velocity at VO2max" OR „velocity associated with VO2max" OR vVO2max OR „max* sprinting speed“ OR „max* speed“ OR MSS OR „max* velocity“ OR „peak speed“ OR „anaerobic speed reserve“ OR “anaerobic velocity reserve” OR ASR)

AND

(sport* OR athlete OR „team sport“ OR „individual sport“)
